# Elucidation of Analytical–Compositional Fingerprinting of Three Different Species of Chili Pepper by Using Headspace Solid-Phase Microextraction Coupled with Gas Chromatography–Mass Spectrometry Analysis, and Sensory Profile Evaluation

**DOI:** 10.3390/molecules27072355

**Published:** 2022-04-06

**Authors:** Emanuela Trovato, Federica Vento, Donato Creti, Paola Dugo, Luigi Mondello

**Affiliations:** 1 Department of Chemical, Biological, Pharmaceutical and Environmental Sciences, University of Messina, Viale Annunziata, 98168 Messina, Italy; pdugo@unime.it (P.D.); lmondello@unime.it (L.M.); 2 Chromaleont s.r.l., c/o Department of Chemical, Biological, Pharmaceutical and Environmental Sciences, University of Messina, Viale Annunziata, 98168 Messina, Italy; federica.vento@chromaleont.it; 3 Mediterranean Food Science, Lungarno della Zecca Vecchia 28, 50122 Firenze, Italy; donato.creti@medfoodscience.com; 4 Department of Sciences and Technologies for Human and Environment, University Campus Bio-Medico of Rome, Via Alvaro del Portillo 21, 00128 Rome, Italy

**Keywords:** solid-phase microextraction (SPME), GC-MS, LRI, volatile fraction, chili peppers, food, marker, principal component analysis (PCA), hierarchical cluster analysis (HCA), EVOOs, sensory profile

## Abstract

The aim of the present study was to determine the volatile compounds of three different species of chili peppers, using solid-phase microextraction (SPME) methods in combination with gas chromatography–mass spectrometry (GC-MS). The detection of marker aroma compounds could be used as a parameter to differentiate between species of chili peppers for their detection and traceability in chili pepper food. The sensorial contribution was also investigated to identify the predominant notes in each species and to evaluate how they can influence the overall aroma. Three different pepper species belonging to the *Capsicum genus* were analyzed: *Chinense*, *Annuum,* and *Baccatum.* A total of 269 volatile compounds were identified in these species of chili peppers. The *Capsicum annum* species were characterized by a high number of acids and ketones, while the *Capsicum chinense* and *Capsicum baccatum* were characterized by esters and aldehydes, respectively. The volatile profile of extra virgin olive oils (EVOOs) flavored with chili peppers was also investigated, and principal component analysis (PCA) and hierarchical cluster analysis (HCA) of the volatile profiles were demonstrated to be a powerful analytical strategy for building a model that highlights the potential of a volatile characterization approach for use in evaluating food traceability and authenticity.

## 1. Introduction

Chili peppers are used as food or spice and are widely used by the food industry as an ingredient for different kinds of flavored and spiced products. The *genus Capsicum* comprises five species: *Capsicum annuum* (containing NuMex, Jalapeno, and Bell varieties), *Capsicum frutescens* (containing the Tabasco variety), *Capsicum chinense* (containing the Habanero and Scotch Bonnet varieties), *Capsicum baccatum* (containing the Aji varieties), and *Capsicum pubescens* (containing the Rocoto and Manzano varieties) [1]. Even though the volatile profile of chili peppers *belonging* to the *Capsicum annuum* species has been analyzed, the volatile profile of *C. baccatum* has not been well investigated [2]. Furthermore, volatile compounds in *C. chinense* were identified and quantified as in previous articles [3,4,5,6,7,8], but no direct comparison between the volatile profile of different *C. chinense* peppers and other pepper species has been made.

The aim of the present study was to analyze, by using solid-phase microextraction–gas chromatography (SPME-GC), the volatile profiles of 17 pepper varieties belonging to three of the five major cultivated species to assess the quality of the analyzed cultivars and to determine the marker compounds responsible for their aromatic characteristics and thus identify them as additives in food products.

Most of the studies presented in the literature were limited to the identification of volatiles without any sensorial tests, and therefore, the real contribution of individual compounds to the overall aroma has not been accurately established. In this respect, the sensory profile of chili peppers was also investigated in order to find a correlation with volatile components and to evaluate which notes contribute the most to the perceived aroma.

Furthermore, the volatile profile of chili-pepper-flavored foods has not been investigated in depth, and the results regarding the capsaicinoids content have mostly been investigated. For this reason, the volatile profile of three commercial extra virgin olive oils (EVOOs) flavored with chili pepper was examined by gas chromatographic analysis, comparing their profile with that of conventional unflavored extra virgin oils.

The linear retention index (LRI) system was used as a supplementary tool for the recognition of the compounds in combination with the mass spectra, and it made possible the reliable identification and accurate quantification of volatiles in chili peppers and chili-pepper-flavored olive oils.

## 2. Results and Discussion

### 2.1. Samples Analyzed

A total of seventeen fresh chili pepper samples belonging to the genus *Capsicum* were collected at the same growth phase in their full ripening stage and kindly provided by Azienda Agricola Salvadori Rita (Livorno, Italy). After their arrival, all the chili peppers were frozen until the day of the analysis and analyzed within two weeks from the freezing process. For each species, three whole chili peppers were ground together, and four homogenized samples were weighted in SPME vials, one for GC-MS and three for GC-FID analysis, assuming the analytes identified were a mean of their content in each pepper. The vials were closed and put in the fridge until the analyses, which were carried out consecutively on the same day. Three chili pepper extra virgin olive oils were purchased online. From the information reported on the label, one of them was flavored with Merkén pepper (a smoked Aji chili pepper), the second was flavored with a mix of *Capsicum chinense* peppers, and the third was flavored with the addition of a mix of different chili peppers. For convenience, the three extra virgin flavored olive oils samples were called EVOO1, EVOO2, and EVOO3, respectively. Table 1 lists the investigated samples.

### 2.2. Volatile Fraction Analysis

**Figure 1:**
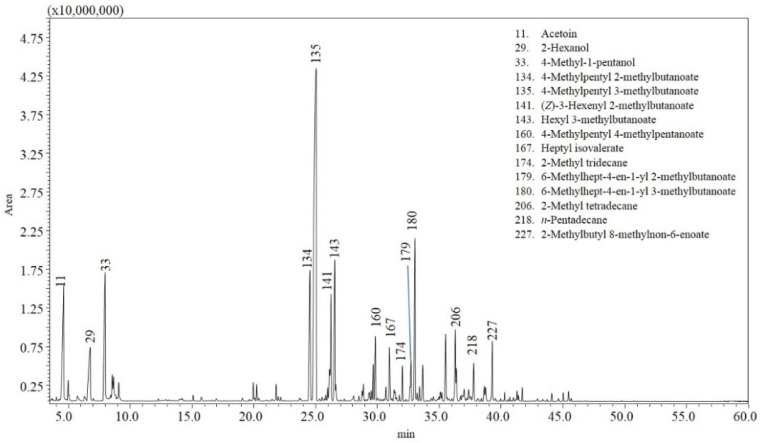
GC-MS analysis of the volatile profile for sample 1 (*C. chinense*—Naga Morich).

**Figure 2:**
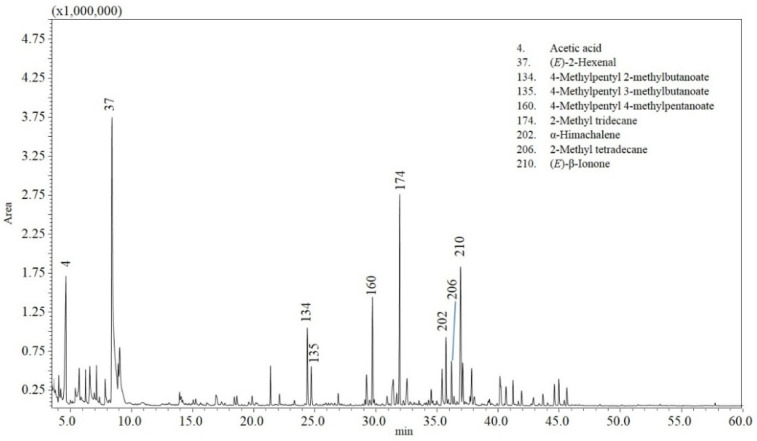
GC-MS analysis of the volatile profile for sample 14 (*C. annuum*—Calabrian pepper).

**Figure 3:**
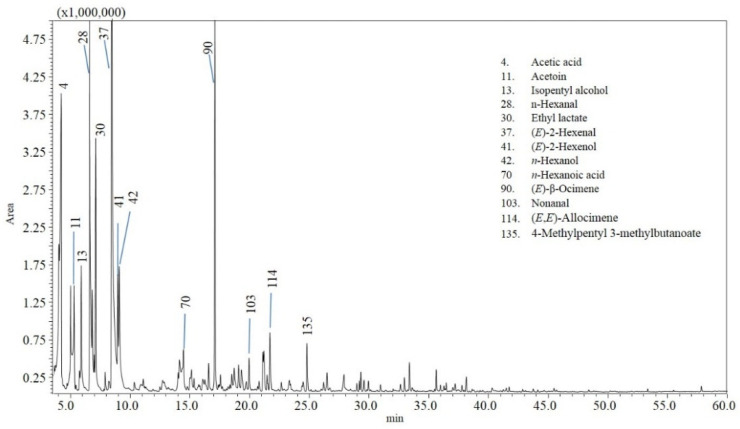
GC-MS analysis of the volatile profile for sample 17 (*C. baccatum*—Aji).

The analyzed chili samples displayed different gas chromatography–mass spectrometry (GC-MS) chromatograms. Figure 1, Figure 2 and Figure 3 show an example of a chromatogram for each variety. More than two hundred and fifty compounds were identified in total in the different samples, accounting for 87–91% of the total composition (Table 2 and Table S1 from Supplementary Materials). Table 2 reports volatile compounds with a percentage area greater than 0.5% in at least one of the chili peppers samples analyzed, and Table S1 from Supplementary Materials reports the other volatile compounds with a percentage area less than 0.5%.

Volatile organic compounds (VOC) are commonly correlated with food flavor and fragrance, and their determination is important to evaluate food quality, authenticity, purity, and origin [9]. Methyl and ethyl esters, for example, provide strong fruity notes in foods, whereas terpenes provide woody, floral, fruity, and spicy notes. Aldehydes have a low odor threshold, and the sniffing analysis of *Capsicum* reported their presence as green, cucumber, pungent, or herbaceous odor notes [10,11].

In addition, it has been proven that the volatiles’ profile of *Capsicum* is mainly affected by varieties [5], ripening stages [3], and processing [12].

The analyses carried out on *Capsicum chinense* peppers revealed that the most relevant compounds are esters (4-methylpentyl 3-methylbutanoate, 6-methylhept-4-en-1-yl 3-methylbutanoate, 4-methylpentyl 2-methylbutanoate, hexyl 3-methylbutanoate, (*Z*)-3-hexenyl 2-methylbutanoate, 2-methylbutyl 8-methylnon-6-enoate, 6-methylhept-4-en-1-yl 2-methylbutanoate, heptyl isovalerate, 4-methylpentyl 4-methylpentanoate, 6-methylheptyl 3-methylbutanoate, and (*Z*)-3-hexenyl 3-methylbutanoate) (Figure 1).

The presence of several aliphatic esters in the *C. chinense* variety has been reported in the literature [3,4,5,6]; it has been confirmed that esters, especially straight-chain esters, are generally metabolized from fatty acids through oxidation [13], and that branched saturated and non-saturated esters can be derived from amino acids’ metabolism [5].

**Figure 4:**
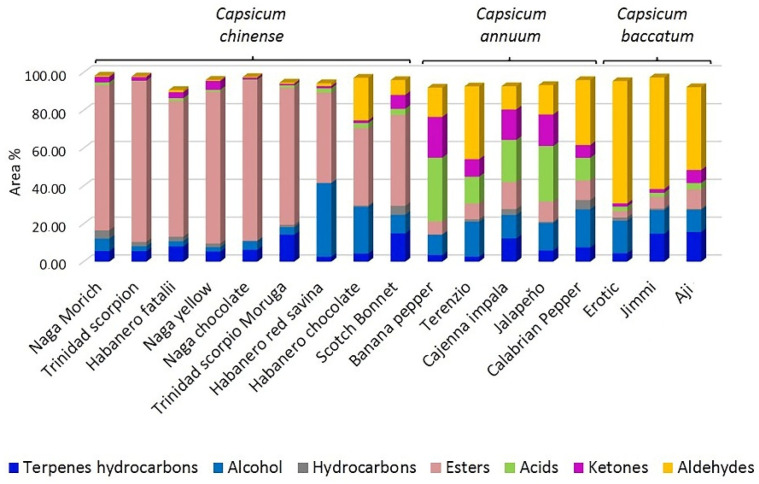
Distribution of volatile compounds’ class identified in the analyzed samples.

Among the analyzed *C. chinense* samples, Habanero red savina, Habanero chocolate, and Scotch bonnet showed a lower content of esters and a greater amount of alcohols and aldehydes (Figure 4). It has been demonstrated that ester biosynthesis is limited by alcohol concentration, which can modify the content of esters in specific cultivars [14]. In the analyzed species, even if there is good alcohol availability, the production of the esters is probably inhibited by the absence of free fatty acids.

Literature data on the *Capsicum Chinese* variety [3,4] report the presence of a little amount of 2-isobutyl-3-methoxypyrazine in the Habanero peppers variety (0.01 mg kg^−1^). Effectively, the three Habanero chili peppers analyzed in the present study did not present this compound (Habanero fatalii (sample 3) and Habanero red savina (sample 7)), or had a very little quantity (Habanero chocolate (sample 8) (0.06%)) of 2-isobutyl-3-methoxypyrazine, which was mainly detected in the Scotch Bonnet variety (sample 9) (0.52%).

The volatile profile of the analyzed *Capsicum annuum* samples was principally characterized by acids, in particular acetic; aldehydes ((*E*)-2-hexenal, *n*-hexanal, 2-methylbutyraldehyde, and isovaleric aldehyde); ketones (acetoin and 3-methyl-2-butanone); alcohols (4-methyl-1-pentanol and isoprenol); and esters (4-methylpentyl 3-methylbutanoate and 4-methylpentyl 2-methylbutanoate) (Table 2 and Table S1 and Figure 2 and Figure 4).

Terenzio and Calabrian pepper varieties showed a greater abundance of (*E*)-2-hexenal than the others belonging to the same species. Only the Jalapeňo pepper contained 6-Methylhept-4-en-1-yl 2-methylbutanoate and 6-Methylhept-4-en-1-yl 3-methylbutanoate.

Banana and the Jalapeňo chili pepper showed the highest amount of acids, followed by Cayenna impala, Terenzio, and Calabrian varieties.

Regarding alcohols, the Calabrian pepper is the only one distinguished by a great percentage of (*E*)-3-hexenol (8.99%).

Concerning terpenoids, the Banana pepper showed the highest amount of (*E*)-β-ocimene, which is absent in the Calabrian pepper. The latter, however, has a higher content of α-longipinene, which is absent in Banana and Cayenna pepper varieties. The Cayenna pepper presented a high amount of α-copaene and β-chamigrene. The latter was also found in larger amounts in the Calabrian pepper. The Jalapeňo chili pepper is the only one with an amount of (*E*)-α-bergamotene greater than 1%.

Furthermore, the Cayenna Impala variety presented a higher amount of 2-isobutyl-3-methoxypyrazine than the other samples. This compound was found to possess an extremely potent odor (odor threshold of 2 × 10^−6^ mg kg^−1^), similar to that of fresh green bell peppers [15].

Regarding *Capsicum baccatum* chili peppers, a great contribution to the volatile profile is given by alcohols (*n*-hexanol, (*E*)-3-hexenol, (*E*)-2-hexenol, (*Z*)-2-buten-1-ol); aldehydes ((*E*)-2-hexenal, *n*-hexanal, 4-methyl-2-pentenal); and esters (ethyl hexanoate, isobutyl 8-methylnon-6-enoate, 4-methylpentyl 3-methylbutanoate, 4-methylpentyl 3-methylbutanoate, ethyl lactate) (Table 2, Table S1 and Figure 3). 

The Aji variety has higher ketones, esters, and terpenes contents than the other chili peppers of the same species. The high percentage area encountered for terpenes, ketones, and esters is relative to (*E*)-β-ocimene, 3-pentenone, and isopropyl acetate, respectively. The Erotic and Jimmi varieties are similar in respect to the amount of aldehydes, ketones, and hydrocarbons identified.

Compounds, such as α-ionone and β-ionone, which may be formed by oxidative degradation of δ-carotene, β-carotene, and terpenoids [16], were particularly found in orange peppers belonging to this third species.

Although the literature reports several studies on the aroma and the content of capsaicinoids in chili peppers [17], to the best of our knowledge, capsaicinoids [18,19] and the volatile profile of chili-pepper-flavored extra virgin olive oil have not been investigated well [19]. For this reason, a study on the aroma profile of flavored EVOOs was carried out and compared to that of fresh peppers and unflavored olive oils. Table S2 reports the results of the volatile compounds identified in the flavored olive oils.

### 2.3. Statistical Analysis

**Figure 5:**
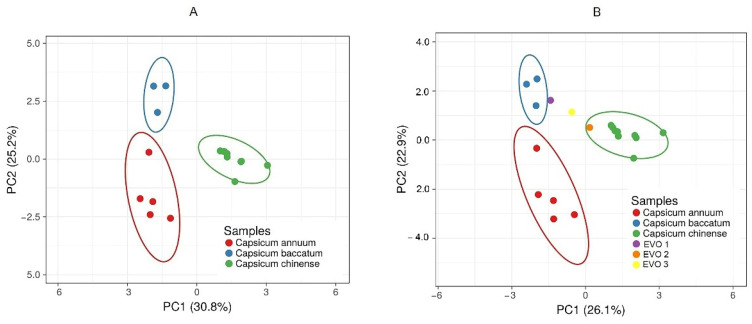
PCA analysis based on relative percentage areas of the 118 most abundant identified volatiles in chili peppers (**A**) and in chili peppers compared with chili-pepper-flavored extra virgin oils (**B**). *X* and *Y* axis show principal component 1 and principal component 2, which explain (**A**) 30.8% and 25.2% of the total variance and (**B**) 26.1% and 22.9% of the total variance, respectively. Prediction ellipses are such that with probability of 0.95, a new observation from the same group will fall inside the ellipse. *N* = 19 data points.

PCA was performed on the 118 most abundant volatile compounds identified in chili peppers (Figure 5A) and on the same numbers of volatiles, also including the data acquired for chili-pepper-flavored extra virgin olive oils (Figure 5B). For statistical data treatment, the following conditions were applied: original values are ln(x)-transformed; rows are centered; Pareto scaling is applied to rows; SVD with imputation is used to calculate principal components. 

As shown in Figure 5A, the PCA score plot in the space of the two PCAs explains 56.0% of the total variance, only considering the peppers, and 49.0% of the total variance, also considering the oils (Figure 5B). This confirms the applicability of the built model and the other unknown samples. From Figure 5, it is clear that at positive values of PC1 and negative values of PC2, the *Capsicum baccatum* species is well separated, whereas the *Capsicum annuum* is present at negative values of PC1 and PC2. Furthermore, the *Capsicum chinense* species is separated well on PC1 in the positive region. As far as the PC2 shown in Figure 5B is concerned, it is interesting to notice that the extra virgin olive oils EVOO1 (containing Merkén pepper, a smoked Aji chili pepper belonging to *Capsicum baccatum*) and EVOO2 (containing a mix of the *Capsicum chinense* pepper) are correctly grouped with the peppers used as a flavoring in the producing process. Since the EVOO3 samples were flavored with a mix of *Capsicum* belonging to different species, it is not possible to insert them into a specific group. Table S4 from Supplementary Materials lists the compounds that mainly influence the plot and their contribution to PC1 and PC2.

Hierarchical cluster analysis (HCA) was performed using both the relative percentage area of the class of the compound identified (Figure S1 from Supplementary Materials) and the relative percentage area of the most abundant volatiles (118) (Figure S2 from Supplementary Materials).

The cluster analysis based on the identified compounds class showed an overlap between the different species; consequently, it is not possible to distinguish between different *Capsicum* species by only considering their contribution (Figure S1 from Supplementary Materials).

The cluster analysis based on the patterns of the most abundant volatiles instead showed good separation of *C. chinense* from the *C. annuum* and *C. baccatum* group (Figure S2 from Supplementary Materials).

**Figure 6:**
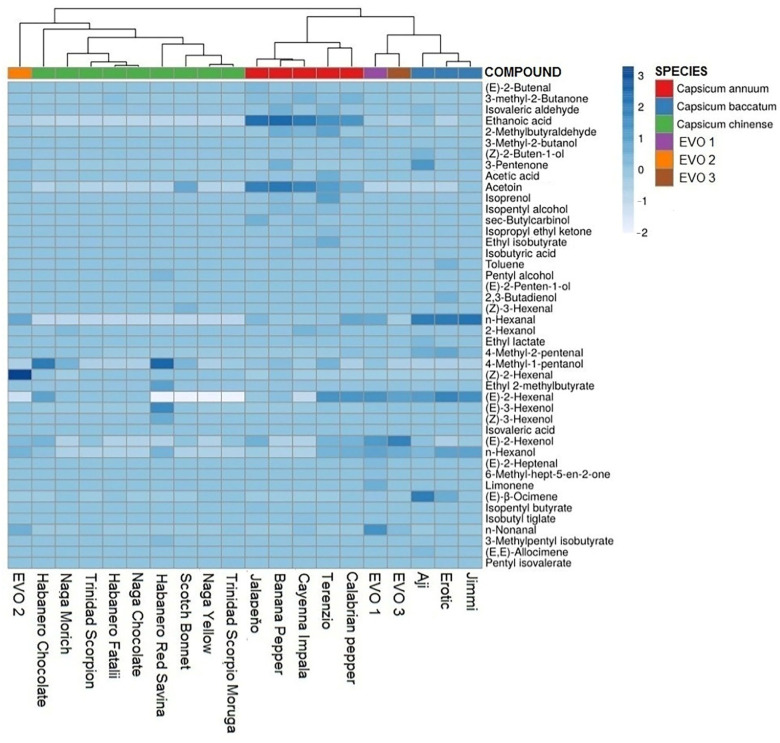
Hierarchical cluster analysis based on relative percentage areas of the 118 most abundant identified volatiles. Original values are ln(x + 1)-transformed. Rows are centered; Pareto scaling is applied to rows. Imputation is used for missing value estimation. Columns are clustered using correlation distance and average linkage. There is a total of 44 rows and 20 columns.

In accordance with PCA analysis, HCA built by introducing the volatile patterns of the three flavored olive oils shows the EVOO2 sample grouped with *Capsicum chinense* peppers and EVOO1 grouped with the *Capsicum baccatum* species, confirming the goodness of the model (Figure 6). In addition, the results confirm the information on the labels of EVOO1 and EVOO2 and guarantee the quality of commercial products, confirming the usefulness of the model for this purpose.

### 2.4. Sensorial Analysis

With regard to the aroma, the sensorial test revealed a wide range of odor impressions in the 17 varieties of chili peppers examined.

**Figure 7:**
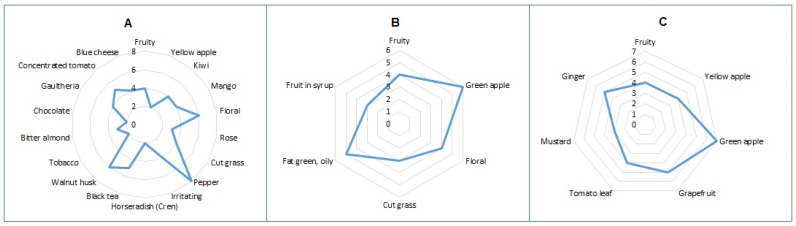
Aroma profile of three different species of pepper chosen as example from descriptive sensory analysis on line scale (*n* = 10). Panel (**A**) shows sample 1 (Naga Morich, *Capsicum chinense*); panel (**B**) shows sample 14 (Calabrian pepper, *Capsicum annuum*); and panel (**C**) shows sample 17 (Aji, *Capsicum baccatum*).

The chili peppers belonging to the *C. chinense* species were mainly characterized by exotic, fruity, and/or sweet notes (Figure 7 and Figure S3 from Supplementary Materials), and their aromas were the most intense among all samples investigated. Such notes are due to the presence of numerous esters, especially 4-methylpentyl 3-methylbutanoate and 4-methylpentyl 2-methylbutanoate [20], Hexyl 3-methylbutyrate, (*Z*)-3-Hexenyl 3-methylbutyrate, and (*Z*)-3-Hexenyl 2-methylbutyrate. The dairy, buttery, and creamy notes found in some peppers, such as Naga morich, Habanero fatalii, Naga chocolate, Trinidad scorpio moruga yellow, Habanero red savina, Scotch bonnet, and Habanero chocolate, are due to the presence of some ketones such as acetoin [21,22], and especially medium-short chain fatty acids such as *n*-decanoic, which characterizes the base note of the Habanero fatalii.

The sweet note of vanilla perceived in the Scotch Bonnet is confirmed from an analytical point of view by the presence of guaiacol, a compound that characterizes the vanilla beans [21,22].

Similarly, the note of “wintergreen” (*Gaultheria procumbens*) found in the Scotch Bonnet and Habanero Fatalii is confirmed by the presence of methyl salicylate in the volatile profile [21,22].

In contrast, *C. annuum* and *C. baccatum* showed different intensity and aroma profiles with a medium-intense mixture of fruity and vegetable-like notes (Figure 7 and Figures S4 and S5 from Supplementary Materials) due to the presence of (*E*)-2-hexenal in both species, and also depending on isopentyl alcohol and *n*-hexanol in *C. annuum and C. baccatum*, respectively.

The persisting final perceived note of cheese in *C. annuum* is dependent on *n*-isovaleric acid [21,22]. Additionally, in some chili peppers belonging to *C. baccatum,* such as the Banana pepper and Cayenna impala, dairy and blue cheese notes were found due to the presence of *n*-hexanoic acid, but they do not really affect the perceived aroma on the olfactory level.

In the peppers belonging to the *C. baccatum* species, the fruity notes are predominant, in particular the green and yellow apple typical of (*E*)-2-hexenal and pentyl isovalerate [21,22], respectively, and the perceived aroma is generally light.

## 3. Materials and Methods

### 3.1. Standard Compounds (Reagents)

A C7–C40 Saturated Alkanes (1000 μg/mL) standard mixture in hexane (49452-U) supplied by Merck Life Science (Darmstadt, Germany) was utilized for ALKANEs linear retention indices (LRIs) calculation. Forty-seven standard compounds (Table S3 from Supplementary Materials) supplied by Merck Life Science (Darmstadt, Germany) were used for the training of the panelist for sensory analysis.

### 3.2. SPME Extraction Conditions

For the method optimization, two SPME fibers supplied by Merck Life Science (Darmstadt, Germany) were tested: carboxen/polydimethylsiloxane (CAR/PDMS), 75 μm 1 cm long (57343-U), and divinylbenzene/carboxen/polydimethylsiloxane (DVB/CAR/PDMS), 50/30 μm 1 cm long (57329-U). The fibers were conditioned before the initial use according to manufacturer’s instructions, and a cleaning step of 20 min at 10 °C below fiber recommended maximum temperature was applied between consecutive analyses. GC analyses were carried out using for each test a 10 mL vial with 0.2, 0.3, and 0.4 g of ground sample, respectively, inserting the fiber 2 cm above the sample, and the best results were obtained for a 0.2 g sample weight.

Four different fiber exposure times were tested: 30, 40, 50, and 60 min. The highest volatile extraction yield was achieved after an exposure time of 50 min, and most of the heavier molecular weight volatiles remained substantially stable thereafter. Furthermore, a sample conditioning time of 5 and 10 min was evaluated at the same temperature (30 °C, 40 °C, 50 °C, or 60 °C) employed for the extraction stage, and the analytical repeatability was excellent in both conditions. Different stirring rates (200 and 300 rpm) for sample conditioning and extraction were also investigated. 

In this investigation, the (DVB/CAR/PDMS) 50/30 μm fiber was found to be the most useful in covering the wide range of chili pepper volatile analytes; a conditioning time of 5 min and an extraction temperature of 50 °C were the best compromise between equilibration time and method sensitivity. Furthermore, a time of 50 min at the same temperature and stirring rate of 300 rpm were proven to be the best choices for an exhaustive extraction of the volatiles components (Figures S6–S10 from Supplementary Materials show method optimization). 

The same extraction condition was adopted for the flavored extra virgin olive oils, using 1 mL as sample volume.

After the extraction, the analytes were manually injected in splitless mode and thermally desorbed for 1 min at 260 °C in the GC injector port.

### 3.3. GC–MS and GC-FID Analysis

GC-MS and GC-FID analyses were carried out for qualitative and quantitative purposes, respectively.

GC-MS analyses were carried out on a GC-QP2020 system (Shimadzu, Kyoto, Japan). For the separation, an SLB-5 ms fused-silica capillary column (30 m × 0.25 mm i.d. × 0.25 μm df) (29804-U) (Merck KGaA, Darmstadt, Germany) was applied. Helium was used as carrier gas at a constant linear velocity of 30.0 cm/s, which corresponded to an inlet pressure of 24.2 kPa. An inlet liner, direct SPME type, straight design unpacked (2633501) (Merck KGaA, Darmstadt, Germany) was used. The injector was equipped with a Thermogreen LB-2 Septa, plug (20608) (Merck KGaA, Darmstadt, Germany), and the temperature was set at 260 °C. The temperature program was the following: 40 °C, held for 1 min, to 350 °C at 3 °C/min, held for 5 min. The interface and ion source temperatures were 250 °C and 200 °C, respectively. The acquisition was made in full scan mode in the mass range of 40–500 m/z, with a scanning rate interval of 0.2 s. Data handling was supported by GCMS solution ver. 4.30 software (Shimadzu, Kyoto, Japan). For the characterization, the following databases were used: W11N17 (Wiley11-Nist17, Wiley, Hoboken, NJ, USA; and FFNSC 4.0 (Shimadzu, Kyoto, Japan). The identification was performed applying two filters, namely, spectral similarity match over 85% and linear retention index (LRI) match calculated using a C7–C40 saturated *n*-alkane homolog series with a filter window of ±10 LRI units.

The LRIs were calculated applying the equation proposed by H. Van den Dool and D. J. Kratz (Equation (1)) [23], developed for programmed-temperature retention index calculation.

(1) $$LRI=100\times \left[z+\left(t_{Ri}-t_{Rz}\right)/\left(t_{R\left(Z+1\right)}\;-t_{Rz}\right)\right]$$

GC-FID analyses were carried out on a GC2010 system (Shimadzu, Kyoto, Japan). Column, oven temperature program, and injection parameters were the same as for MS applications. Helium was used as carrier gas at a constant linear velocity of 30.0 cm/s, which corresponded to an inlet pressure of 97.4 kPa. The FID temperature was set at 280 °C (sampling rate 200 ms), and hydrogen and air flows were 40 mL/min and 400 mL/min, respectively. Data were collected by LabSolution software ver. 5.92 (Shimadzu, Kyoto, Japan). Quantitative results were determined as peak area percentage without any correction. Samples were analyzed in triplicates.

### 3.4. Statistical Procedure

Principal components analysis (PCA) bidimensional visualization, as implemented in ClustVis large version 2.0 (https://biit.cs.ut.ee/clustvis_large, accessed on 25 March 2022), was used for showing relationships between compounds classes and metabolites with *Capsicum* chili peppers species, respectively. For these analyses, the compounds classes and metabolite datasets were ln (x + 1) transformed and mean-centered. Pareto scaling was used as a measure for compounds’ classes–species and metabolite–species correlation and for hierarchical clustering analysis (HCA).

### 3.5. Sensorial Evaluation Procedure

Sensory analysis was carried out by a panel of 7 analysts trained to distinguish and describe the aroma characteristics of 47 pure standards (Table S3 from Supplementary Materials). The first step was to carry out a screening of all the chili pepper samples to identify the descriptors. The overall aroma of accessions was defined by about 61 descriptors, divided into 4 macro-areas: fresh fruity and floral notes; fresh vegetable notes; dry vegetable notes; and other notes (miscellaneous), including woody, dairy, spicy, and notes not attributable to the other categories. Figure S11 from Supplementary Materials reports a list of the descriptors identified by the panel test.

For the sensorial analysis, the peppers were chopped one at a time with an immersion blender to reduce them into pieces of 2/3 mm, and the mixture was then placed on a sheet of absorbent paper to drain the moisture. Each panel smelled the preparation for about 30 min in order to identify the top, the middle, and the bottom notes.

The intensity of the previously identified descriptor was judged on a 10-point scale from 1 = weak to 10 = very strong. The radar graphs for each sample were constructed with the positive average values, excluding the values equal to zero, the minimums, and maxima.

## 4. Conclusions

In this paper, the volatile fingerprinting of 17 varieties of chili peppers belonging to *Capsicum chinense*, *Capsicum annuum*, and *Capsicum baccatum* were profiled using an HS-SPME extraction method followed by GC analysis. Previously, *Capsicum baccatum’s* volatile profile was not well investigated. Furthermore, this is the first work in which such a large number of chili peppers belonging to *Capsicum chinense* is analyzed and discussed in detail.

The diversity in aroma found among the studied cultivar, due to qualitative and quantitative differences of the odor-contributing volatiles, was also confirmed by the sniffing test. In particular, the sensory results revealed *C. chinense* chili peppers have fruity/exotic aromas and are characterized by a high contribution of several esters. The aroma found among *C. annuum* is due to different combinations of fruity/exotic and green/vegetable notes. The notes perceived in *Capsicum baccatum* peppers are principally fruity, and their intensity is weak in respect to that of the other pepper species.

Principal components analysis and hierarchical cluster analysis performed using percentage area of the 118 most abundant volatile compounds enabled a model to be built to distinguish between the different *Capsicum* species investigated. In addition, the volatile profile of chili extra virgin olive oil was investigated in order to find a valuable approach providing useful and comprehensive insights to evaluate the impact of chili flavor addition on extra virgin olive oil’s volatile composition, which highlights the use of this approach for evaluating food traceability and authenticity.

## Figures and Tables

**Table 1:** Chili peppers samples (1–17) and flavored chili pepper EVOOs (18–20) analyzed.

**Sample**	**Chili Pepper Type**	**Specie**	**Color**
1	Naga Morich	*Capsicum chinense*	Red
2	Trinidad Scorpion	*Capsicum chinense*	Red
3	Habanero Fatalii	*Capsicum chinense*	Yellow
4	Naga Yellow	*Capsicum chinense*	Yellow
5	Naga Chocolate	*Capsicum chinense*	Brown
6	Trinidad Scorpio Moruga	*Capsicum chinense*	Orange
7	Habanero Red Savina	*Capsicum chinense*	Red
8	Habanero Chocolate	*Capsicum chinense*	Brown
9	Scotch Bonnet	*Capsicum chinense*	Orange
10	Banana Pepper	*Capsicum annuum*	Yellow
11	Terenzio	*Capsicum annuum*	Red
12	Cayenna Impala	*Capsicum annuum*	Red
13	Jalapeňo	*Capsicum annuum*	Red
14	Calabrian pepper	*Capsicum annuum*	Red
15	Erotic	*Capsicum baccatum*	Orange
16	Jimmi	*Capsicum baccatum*	Orange
17	Aji	*Capsicum baccatum*	Yellow
**Oil Sample**	**Chili Pepper type**	**Specie**	**Color**
EVOO 1	Merkén	*Capsicum baccatum*	Yellow
EVOO 2	Mix of peppers	*Capsicum chinense*	Yellow
EVOO 3	Mix of peppers	*Unknown*	Orange

**Table 2:** Most abundant volatile compounds contained in the chili peppers samples analyzed expressed in area % as GC-FID measurement.

	Compound	LRL_ex_	LRL_lib_	*Capsicum chinense*									*Capsicum annum*					*Capsicum baccatum*		
				1	2	3	4	5	6	7	8	9	10	11	12	13	14	15	16	17
1	(*E*)-2-Butenal	619	629	0.46	0.20	0.45	0.38	0.21	0.32	0.2	0.33	0.78	0.4	0.46	1.19	1.39	0.89	0.50	0.41	0.75
2	3-methyl-2-Butanone	655	657	0.34	0.20	1.20	0.24	0.30	0.07	0.56	0.42	0.72	1.27	1.22	1.68	1.27	1.65	0.60	0.39	0.61
3	Isovaleric aldehyde	657	652	0.18	0.10	0.52	0.10	0.09	0.08	0.20	0.10	0.85	1.80	1.51	0.71	1.07	0.11	0.20	0.13	1.29
4	Acetic acid	659	661	1.02	0.09	0.25	0.77	0.30	0.59	0.39	1.14	0.60	30.41	10.5	19.29	25.97	9.46	1.26	0.97	2.38
5	2-Methylbutyraldehyde	664	662	tr	tr	0.07	tr	tr	0.14	0.15	0.06	0.13	2.08	2.97	1.64	0.62	0.25	0.37	0.09	0.06
6	Isopropyl acetate	660	650	0.06			tr	tr					0.65	0.81	0.12	0.44	0.21			0.99
7	3-Methyl-2-butanol	668	674	tr	tr		0.08	tr	tr		0.08	0.12	0.51	0.63	0.52	0.59	1.40	0.81		0.12
8	(*Z*)-2-Buten-1-ol	673	671	tr	tr		tr	tr		0.18	0.12	0.54	0.19	0.28	0.35	0.56	0.26	0.08	1.23	1.61
9	3-Penten-2-one	690	691	tr	tr	0.10	tr	tr	tr	0.13	0.08	0.18	1.99	0.24	0.85	0.12	0.13			4.25
10	Propionic acid	703	698	0.21		0.07	0.06		tr		0.74	0.22	0.70	1.86	0.48	0.19	0.35	0.19	0.06	0.23
11	Acetoin	726	716	1.40	0.10	0.07	0.27	0.13	0.11	0.09	0.59	4.55	17.53	6.16	11.88	13.88	3.65	0.42		0.55
12	Isoprenol	729	724	tr	tr	0.06	tr	tr	0.11	tr	tr	0.20	1.35	3.13	0.44	0.22	0.05	0.18	0.23	0.25
13	Isopentyl alcohol	733	729	0.09	tr	tr	tr	tr	0.05	1.06	0.79	0.25	1.30	1.25	1.12	0.83	0.32	0.29		1.95
14	sec-Butylcarbinol	738	733	tr	tr	tr	tr		0.07	0.65	0.15	0.19	0.95	0.85	1.18	1.78	0.33			0.11
16	Isopropyl ethyl ketone	745	742	tr	tr	0.06	0.07	tr	tr	0.14	0.11			1.16		0.23	0.26		0.07	
17	Ethyl isobutanoate	754	754	tr	tr	0.06	tr	tr	0.1	0.20	0.5	0.44	0.29	2.08	1.40	0.14	0.32	0.32	0.12	0.18
18	Isobutyric acid	761	774	tr	tr	tr	tr	tr		0.11	0.05	0.52	1.11	0.16	0.72	0.06	0.05		0.12	tr
19	Toluene	764	763					tr			0.24			0.38	0.54	0.20	tr	1.70		0.37
20	Pentyl alcohol	763	763	0.07	tr	tr	tr	tr	0.18	1.44	0.74	0.26	0.16	0.28	0.39	0.15	0.84	0.20	0.17	0.26
21	(*E*)-2-Penten-1-ol	766	761	0.12	tr	0.06	tr				1.03	0.36	0.21	0.61	0.53	0.35	0.28		0.32	tr
22	Prenol	770	772	tr	tr	tr	tr	tr	0.07		0.39	0.27	0.30	0.30	0.30	0.16	0.06	0.33	0.16	0.72
23	2,3-Butadienol	788	788	0.10		tr	tr	tr			0.22	0.41	0.05	0.26	0.65	0.18	0.34	1.63	0.06	0.48
24	3-Methylcrotonaldehyde	787	780		tr	tr	tr	tr				0.20	0.11	tr	0.62	0.21				0.05
25	Isopentyl formate	788	791			tr	tr	tr		0.38				0.23	0.28					0.60
27	(*Z*)-3-Hexenal	798	797		tr	tr			tr	tr	0.45	1.46	0.12	0.23	0.63	0.39				
28	*n*-Hexanal	801	801	tr	tr	0.06	tr	tr	tr	tr	0.85	0.79	2.15	2.35	2.25	3.44	3.74	16.26	20.74	16.38
29	2-Hexanol	806	802	1.43				tr	tr	tr	0.75	0.50	0.29	1.32	1.63	0.19		4.47		0.27
30	Ethyl lactate	806	814					tr	tr		0.22	0.21	0.47	0.12	0.39	0.20		0.98	0.71	1.37
31	4-Methyl-2-pentenal	816	814			tr	tr	tr	tr	tr		0.53	0.11	0.37	0.32	0.65	1.32	2.75	1.46	2.04
33	4-Methyl-1-pentanol	838	832	2.72	0.44	0.67	0.40	0.51	1.37	23.15	13.61	2.48	2.03	2.72	1.41	1.59	0.52	0.21	0.19	0.35
35	Ethyl 2-methylbutanoate	846	842	0.06	tr	0.13	0.11	tr	0.11	2.86	0.79	0.49	0.4	0.36	0.16	0.23	0.27			0.14
37	(*E*)-2-Hexenal	850	850				tr		0.09	0.61	19.38	0.59	8.05	29.8	2.46	6.57	26.85	43.22	34.59	21.58
38	(*E*)-3-Hexenol	854	847	0.50	0.53	0.25	0.50	0.49	0.55	6.07	0.68		0.33		0.15	0.88	8.99		3.36	
39	(*Z*)-3-Hexenol	856	856	0.32	0.31	0.15	tr	0.21		2.11	0.54		0.36							
40	Isovaleric acid	838	842	0.11	0.11	0.18	tr	0.05	0.38	0.63	0.41	0.21	0.61	0.87	0.20	1.01	0.77			
41	(*E*)-2-Hexenol	864	864	0.09		0.07	tr	tr		0.36	2.80		1.04	2.89	0.19	3.08	2.91	1.07	1.58	1.90
42	*n*-Hexanol	868	867	0.37	0.74	0.12	0.3	0.57	0.25	2.92	1.89	0.26	0.91	2.74	0.42	1.45	3.22	7.53	3.63	2.44
43	2-Methylbutyric acid	883	881	tr	tr	tr	tr	tr	tr	0.27	0.07	0.11			0.22	0.65	0.63	tr	0.46	0.14
44	*n*-Pentanoic acid	889	918	tr	tr	tr	tr	tr	tr	0.10		0.09	0.20	0.60	0.23	0.44	0.12	tr		tr
60	3-Methyl-ethylpentanoate	962	953	tr	tr	tr	tr	tr	tr	0.64		0.86	0.06	tr	tr	0.10	tr		0.25	tr
69	6-Methyl-hept-5-en-2-one	984	986	tr	tr	0.06	tr	tr	0.07	tr	0.06	0.52	0.48	0.21	0.31	0.57		0.11	0.18	0.46
70	*n*-Hexanoic acid	989	997	tr	tr	0.10	tr	tr	tr	0.07	0.07	0.71	0.34	0.14	0.24	0.67	0.42	0.51	0.36	0.08
71	Ethyl hexanoate	998	1003	tr	tr	0.12	0.06	tr	tr	tr	0.58		0.68		0.08	tr	tr	0.35	0.89	0.52
80	*p*-Cymene	1025	1024	tr	tr	tr	tr	tr	tr	tr	0.33	0.6	0.07	0.15	0.12	0.06	tr	tr	0.33	0.05
81	Limonene	1028	1030	tr	0.06	tr	tr	tr	tr	0.07	0.31	1.05	0.06	0.22	0.55	0.41	tr	0.08	0.18	0.43
84	(*Z*-β-Ocimene	1035	1035	tr			tr		tr	tr	tr	0.23	tr	tr	tr	tr	tr	tr		0.64
90	(*E*)-β-Ocimene	1046	1046		tr			tr		0.17	0.05	0.97	0.96	0.3	0.32	0.24		2.55		9.70
91	Isopentyl butanoate	1048	1050		tr	tr	0.05	tr	tr	0.14	0.3	0.05		tr		1.08	0.06	0.05	0.06	0.06
94	(*E*)-2-Octenal	1067	1058	tr	tr	tr	tr	tr	tr		0.72	0.09	0.17	0.06	0.11	0.07	tr	0.06	0.07	tr
98	Guaiacol	1086	1094	tr		tr	tr	tr	tr	tr	0.13	0.70	tr	tr		0.07	tr	0.10	0.38	0.52
99	Isobutyl tiglate	1091	1093	tr	tr	tr	tr	tr	tr	0.08	tr	0.20	0.12	tr	1.13	0.20	tr	0.19	0.34	0.64
100	3-Methylbutyl 2-methylbutanoate	1098	1104	tr	tr	tr	tr	tr	0.22	tr	0.19		0.10		0.13	tr		0.09	tr	0.54
101	Linalool	1098	1101		tr	tr	tr	tr						0.30	0.83	0.17	0.06	0.08	0.19	tr
103	*n*-Nonanal	1103	1107	tr		tr	tr	tr		tr	tr	0.47	0.11	0.18	1.33	0.25	0.25		0.21	0.75
104	3-Methylbutyl isovalerate	1104	1109	0.26	0.07	0.29	0.29	0.09	0.92	0.25	0.35	0.86	0.27	0.29		0.67				
105	2-Methylbutyl isovalerate	1106	1109	tr		tr	tr	tr	0.15	tr	0.65		tr		0.09	tr	0.11		tr	tr
106	3-Methylpentyl isobutanoate	1110	1115	0.15	tr	0.29	0.15	0.06	0.32	2.08	0.05	0.21	0.06	0.06	0.70	0.57	tr	tr	0.47	tr
107	Isohexyl isobutanoate	1112	1110	tr	0.75	tr	tr	tr	tr	tr	0.09	0.79	tr	0.22	0.12	0.14	tr	0.07	tr	tr
111	(4*E*,6*Z*)-Alloocimene	1128	1128	tr		tr			tr	tr		0.07			0.07	0.09	tr	0.13	tr	0.90
112	2-Vinylanisole	1130	1135						tr	tr	tr	0.08	tr	tr		0.09		0.15		0.87
114	(*E*,*E*)-Allocimene	1140	1145					tr				0.11		0.05		0.06	tr	0.21	0.08	1.33
116	Pentyl isovalerate	1142	1143	0.34	0.18	0.23	0.64	0.08	1.08	0.21	0.27	0.07	tr		0.10					tr
117	Hexyl isobutanoate	1146	1150	tr	0.54	0.13	0.13	0.06	0.12	0.25	0.05	0.14	tr	0.06	0.14		tr			tr
125	3-Methoxy-2-isobutylpyrazine	1175	1176							tr	0.06	0.52	0.19	tr	1.74	0.07	tr	tr	0.50	
134	4-Methylpentyl 2-methylbutanoate	1198	1202	6.54	3.14	2.41	2.33	1.08	9.80	13.21	12.86	4.58	1.31	1.40	1.23	1.12	2.95	0.05	0.13	0.31
135	4-Methylpentyl 3-methylbutanoate	1209	1206	33.12	21.11	16.02	21.67	13.98	32.8	22.69	16.65	20.13	2.32	1.44	2.21	1.42	1.52	0.62	0.76	1.16
138	Citronellol	1222	1232	0.11		tr	0.07	1.99	tr	tr	0.11	0.07	tr	tr	tr	tr	tr	tr		tr
140	ESTER	1229		0.78	1.19	0.55	0.64	0.74	0.25	0.32	1.17	0.12	0.07	tr	0.11	tr	tr	tr	tr	0.16
141	(*Z*)-3-Hexenyl 2-methylbutanoate	1233	1231	2.41	4.79	2.36	1.34	2.02	1.59	1.64	0.89	0.48	tr	0.05	0.20	0.05	tr	tr	tr	tr
142	(*Z*)-3-Hexenyl 3-methylbutanoate	1237	1243	1.02	2.92	1.42	7.74	1.50	9.55	0.96	0.57	0.65	tr	0.05	0.17	0.11	tr	tr	0.05	0.31
143	Hexyl 3-methylbutanoate	1246	1243	4.02	7.88	4.57	12.34	8.87	7.77	0.16	0.09	0.17	tr	tr	0.10	tr	tr	tr	0.12	tr
144	(E)-Hex-2-enyl 3-methylbutanoate	1248	1243	0.37	0.45	0.18	0.32	0.96	1.14	tr	tr	0.06		tr	0.12	tr	0.21	tr	tr	tr
145	Heptyl isobutanoate	1251	1248	tr	0.80	0.21	0.17	tr	0.07	tr	tr	tr		tr	0.09	tr	tr	tr		tr
151	6-Methylhept-4-en-1-yl isobutanoate	1289	1293	0.15	1.39	0.79	0.09	0.99	0.05	0.05	tr	0.09	tr	tr	0.08			tr		tr
152	3-Methylpentyl (2*E*)-2-methyl-2-butenoate	1291	1300	0.5	0.39	0.17	0.39	0.2	0.47	0.14	1.77	0.22			0.09	tr	tr	tr		0.05
155	5-Methylhexyl 3-methylbutanoate	1300	1303	0.14		0.21	0.35		tr	0.06	0.82	1.86	1.09	0.54	2.39	0.31	0.62	0.08	0.19	0.20
157	ESTER	1302		0.17	0.23	0.07	0.13		0.17	0.11	0.66			tr		tr				
158	Heptyl butanoate	1308	1298	0.18	0.24	0.41	1.16	0.31	0.15	0.10	0.12					tr				tr
159	4-Methylhexyl 2-methylbutanoate	1308	1307	0.87	0.62	0.33	0.12	0.12	0.44	0.38	0.27	0.12	0.06		0.11	0.10	tr		0.05	
160	4-Methylpentyl 4-methylpentanoate	1313	1315	1.63	1.09	1.02	0.64	0.76	0.46	0.60	0.53	0.27	tr	0.07	0.08	tr	2.02	tr	tr	tr
165	Heptyl 2-methylbutanoate	1332	1333	0.31	1.45	0.59	0.66	0.91	0.14	tr	tr	0.29	tr		tr	tr				tr
167	Heptyl isovalerate	1338	1338	1.67	4.97	3.38	4.09	5.50	0.46	0.13	0.08		tr	tr	0.26	tr	tr	tr	0.05	0.11
170	α-Cubebene	1347	1347	0.68	0.31	tr	0.69	0.48	0.21	0.06		0.09	tr	tr	0.08	tr		tr	0.07	
171	ESTER	1352		0.24	0.75	0.3	0.39	0.57	0.12	0.1	tr	0.09		tr	0.07	tr				tr
172	α-Longipinene	1354	1352	0.08	0.06	0.34		0.35	tr	0.05	tr	1.57		tr		0.09	1.01			tr
174	2-Methyl tridecane	1362	1365	1.54	0.21	0.19	0.31	0.16	0.16	0.05	0.07	1.26	0.05	0.21	0.53	0.33	3.28	tr	0.28	tr
176	α-Ylangene	1372	1371	0.09	tr	tr	tr	1.43	0.07	tr	-			tr	0.06		tr	tr		tr
177	Cyclosativene	1370	1367			tr	tr			tr	0.05	0.58	0.06	tr	0.17	tr		tr		tr
178	α-Copaene	1376	1375	tr	0.73	tr	0.73		0.06	0.08	0.27		0.18	0.34	1.83	0.56	0.65	tr	0.36	0.09
179	6-Methylhept-4-en-1-yl 2-methylbutanoate	1378	1383	1.72	1.61	2.02	1.45	16.3	0.44	0.05	0.09	10.37				1.84	0.07		0.56	tr
180	6-Methylhept-4-en-1-yl 3-methylbutanoate	1385	1388	7.77	14.12	11.67	14.45	16.15	1.13	0.81	tr	0.34		0.05	0.19	1.76	tr	tr	tr	0.22
181	β-Elemene	1389	1390	0.22	tr	0.09			0.08	tr		0.59	tr	0.31	0.29	0.19	0.38	0.28	9.36	tr
182	Sativene	1392	1394						tr	tr	tr	0.22	0.07	tr	0.13	0.61		tr		
183	6-Methylheptyl 2-methylbutanoate	1394	1398	0.43	0.77	0.64	0.36	0.57	0.24	tr	tr					tr	tr	tr		0.16
186	6-Methylheptyl 3-methylbutanoate	1399	1402	1.53	1.75	2.10	1.15	3.16	tr	tr	-	0.13				tr	tr	0.06	tr	tr
192	(*E*)-α-Ionone	1421	1421		0.11		0.30	0.24	0.13	tr	tr			tr	0.09		tr	0.52	0.96	
196	(*E*)-α-Bergamotene	1435	1432	tr				0.20	tr	tr	tr			tr		1.12	0.07			tr
197	6-Methyl-4-heptenyl pentanoate	1436	1438	0.09	0.55	0.50	0.60	1.21	0.09	tr	0.16							tr		tr
198	Octyl isovalerate	1437	1441	0.31	0.81	0.58	0.58	1.55	tr	tr	-			tr	tr	tr				tr
199	ESTER	1443		4.19	1.83	0.84	1.10	0.68	0.74	0.11	0.16	1.57	tr	0.20	0.26	0.14	0.51	tr	0.13	tr
200	(*E*)-Geranylacetone	1446	1450	0.10	0.23	0.16	0.06	0.23		tr	tr	0.74	tr	tr	0.11	0.08	tr	tr	0.10	0.27
202	α-Himachalene	1450	1449	0.12	0.10	0.83	tr	0.26			0.45					0.17			0.23	
206	2-Methyl tetradecane	1462	1463	1.11	0.67	1.08	0.77	0.05	0.42	0.07	0.22	1.39	0.09	0.21	0.27	0.11	1.04	tr	0.14	tr
207	Oxacyclododecan-2-one	1467		0.44	0.24	0.93	2.78	0.05	0.09	tr	tr				0.16	tr	0.08		0.09	0.06
210	(*E*)-β-Ionone	1482	1482	0.35	0.74	0.15	0.24	0.14	0.40	0.09	0.06		tr	tr	0.09	0.18	0.21	tr	0.17	tr
211	γ-Himachalene	1483	1481	0.35	0.99	4.74	0.27	0.60	11.64	tr	1.07	1.84	tr	tr	0.09	tr	tr	tr		tr
212	β-Chamigrene	1484	1479	0.41	tr	tr	0.05	0.13	0.30	tr	0.20	0.57	0.06	tr	0.97	0.11	1.40	tr	0.60	
213	6-Methylhept-4-en-1-yl 2-methylbutanoate	1478	1481	0.18		0.32	tr	2.08						0.26	tr	0.22	0.14			0.07
214	Isobutyl 8-methylnon-6-enoate	1488	1496	0.33	0.71	3.36	0.35	0.25	0.10	tr	tr	0.09		tr	0.24	tr	tr	tr	0.86	0.05
217	α-Selinene	1496	1501	0.14	0.50	tr	tr	0.27	0.06	tr	tr					tr			0.78	
218	*n*-Pentadecane	1498	1500	0.54	0.86	0.57	0.39	0.06	0.38	0.06	0.09	0.53	0.06	0.13	0.23	tr	0.05	tr		0.07
219	α-Cuprenene	1501	1508	0.08	tr	0.18	0.11	0.10	0.57	tr	0.21	0.28	0.21	0.15	0.06	0.32	0.31	0.13	0.28	tr
220	Isobutyl 8-methylnonanoate	1502	1496	0.07	0.08	0.39	tr	tr	tr	0.10	tr	1.18			0.74	tr	0.10		tr	0.11
223	δ-Cadinene	1519	1518	0.76	0.59	0.22	0.54	0.30	0.19	tr	tr	0.06		tr	0.10	tr	tr	tr	0.11	tr
227	2-Methylbutyl 8-methylnon-6-enoate	1537	1545	2.14	4.02	6.35	1.19	0.42	0.31	tr	tr		tr	tr	0.11	tr	0.25			tr
235	Dendrolasin	1570	1573	0.24	0.28	0.06	0.55	tr	tr			0.14		tr	0.39	tr	0.09			
237	(*E*)-2-Tridecen-1-ol	1583	1573	0.16	0.15	0.53	0.10	0.16	tr	tr										tr
238	Isopentyl 8-methylnon-6-enoate	1586	1592	0.36	0.27	0.89	0.26	0.96	tr	tr	tr	0.15		tr	0.06	tr	0.80	tr	tr	tr
249	Cadalene	1675	1677	0.12	tr	tr	tr	0.05	tr	tr	tr			tr	0.16	tr	1.40	tr	tr	tr
250	4-Methylpentyl 8-methylnon-6-enoate	1685	1692	0.35	0.28	2.22	0.18	0.15	0.09	tr	tr	0.26				tr	0.19			tr
252	4-Methylpentyl 8-methylnonanoate	1702	1710	0.09	0.07	0.63	0.08	0.05	0.06	tr	0.07	0.08		tr	0.16	0.07	tr	tr		tr
261	ESTER	1852		tr	tr	0.82	tr	tr	tr											
	**Total**			**91.72**	**91.47**	**84.21**	**88.88**	**92.44**	**90.91**	**91.1**	**94.34**	**82.04**	**89.73**	**89.35**	**80.53**	**87.97**	**90.8**	**92.04**	**92.17**	**85.31**

The compound’s number is reported in order of elution, considering the total number of compounds eluted. For the identification of the compounds not reported in this table, see Table S1 from Supplementary Materials. tr = trace compound.

## Data Availability

Not applicable.

## Supplementary Materials

The following supporting information can be downloaded at: https://www.mdpi.com/article/10.3390/molecules27072355/s1, Figure S1: Hierarchical cluster analysis based on the relative percentage areas of identified compound classes; Figure S2: Hierarchical cluster analysis based on the relative percentage areas of the 118 most abundant identified volatiles; Figure S3: Aroma profile of the *Capsicum chinense* pepper from descriptive sensory analysis on the line scale (n = 10); Figure S4: Aroma profile of the *Capsicum annuum* pepper from descriptive sensory analysis on the line scale (n = 10); Figure S5: Aroma profile of the *Capsicum baccatum* pepper from descriptive sensory analysis on the line scale (n = 10); Figure S6: Influence of temperature on SPME method extraction optimization; Figure S7: Influence of time on SPME method extraction optimization; Figure S8: Influence of stirring rate on SPME method extraction optimization; Figure S9: Influence of sample time conditioning on SPME method extraction optimization; Figure S10: Influence of sample volume on SPME method extraction optimization; Figure S11: List of descriptors used in the sensory analysis of chili peppers; Table S1: Less abundant volatile compounds contained in the chili peppers samples analyzed, expressed in area % as a GC-FID measurement; Table S2: Volatile compounds contained in the chili-pepper-flavored olive oil samples analyzed, expressed in area % as a GC-FID measurement result; Table S3: Standard key compounds used for the training of panelists for sensorial analysis; Table S4. Contribution of the variables on PC1 and PC2.

## Sample Availability

Samples from the compounds are not available from the authors upon request.

## References

[1] Pruthi J.S. (1980). Spices and Condiments: Chemistry, Microbiology, Tecnology.

[2] Kollmannsberger H., Rodríguez-Burruezo A., Siegfried Nitz S., Nuez F. (2011). Volatile and capsaicinoid composition of ají (*Capsicum baccatum*) and rocoto (*Capsicum pubescens*), two Andean species of chile peppers. J. Sci. Food Agric..

[3] Pino J., Sauri-Duch E., Marbot R. (2006). Changes in volatile compounds of Habanero chile pepper (*Capsicum chinense* Jack. cv. Habanero) at two ripening stages. Food Chem..

[4] Pino J., González M., Ceballos L., Centurión-Yah A.R., Trujillo-Aguirre J., Latournerie-Moreno L., Sauri-Duch E. (2007). Characterization of total capsaicinoids, colour and volatile compounds of Habanero chilli pepper (*Capsicum chinense* Jack.) cultivars grown in Yucatan. Food Chem..

[5] Pino J., Fuentes V., Barrios O. (2011). Volatile constituents of Cachucha peppers (*Capsicum chinense* Jacq.) grown in Cuba. Food Chem..

[6] Murakami Y., Iwabuchi H., Ohba Y., Fukami H. (2019). Analysis of Volatile Compounds from Chili Peppers and Characterization of Habanero (*Capsicum chinense*) Volatiles. J. Oleo Sci..

[7] Cuevas-Glory L.F., Sosa-Moguel O., Pino J., Sauri-Duch E. (2015). GC–MS Characterization of volatile compounds in Habanero pepper (*Capsicum chinense* Jacq.) by optimization of headspace solid-phase micro-extraction conditions. Food Anal. Methods.

[8] Sosa-Moguel O., Pino J.A., Ayora-Talavera G., Sauri-Duch E., Cuevas-Glory L. (2017). Biological activities of volatile extracts from two varieties of Habanero pepper (*Capsicum chinense* Jacq.). Int. J. Food Prop..

[9] Ko A.-Y., Rahman M.M., El-Aty A.M.A., Jang J., Choi J.H., Mamun M.I.R., Shim J.H. (2014). Identification of volatile organic compounds generated from healthy and infected powdered chili using solvent-free solid injection coupled with GC/MS: Application to adulteration. Food Chem..

[10] Bogusz S.J., Tavares A.M., Teixeira Filho J., Zini C.A., Godoy H.T. (2012). Analysis of the volatile compounds of Brazilian chilli peppers (*Capsicum* spp.) at two stages of maturity by solid phase micro-extraction and gas chromatography-mass spectrometry. Food Res. Int..

[11] RodrÍguez-Burruezo A., Kollmannsberger H., González-Mas M.C., Nitz S., Nuez F. (2010). HS-SPME Comparative Analysis of Genotypic Diversity in the Volatile Fraction and Aroma-Contributing Compounds of Capsicum Fruits from the annuum−chinense−frutescens Complex. J. Agric. Food Chem..

[12] Cremer D.R., Eichner K. (2000). Formation of volatile compounds during heating of spice paprika (*Capsicum annuum*) powder. J. Agric. Food Chem..

[13] Schreier P. (1984). Chromatographic Studies on Biogenesis of Plant Volatiles.

[14] Defilippi B.G., Manríquez D., Luengwilai K., González-Agüero M. (2009). Aroma volatiles: Biosynthesis and mechanisms of modulation during fruit ripening. Adv. Bot. Res..

[15] Luning P.A., de Rijk T., Wichers H.J., Roozen J.P. (1994). Gas Chromatography, Mass Spectrometry, and Sniffing Port Analyses of Volatile Compounds of Fresh Bell Peppers (*Capsicum annuum*) at Different Ripening Stages. J. Agric. Food Chem..

[16] Lewinsohn E., Sitrit Y., Bar E., Azulay Y., Ibdah M., Meir A., Yosef E., Zamir D., Tadmor Y. (2005). Not just colors—Carotenoid degradation as a link between pigmentation and aroma in tomato and watermelon fruit. Trends Food Sci. Technol..

[17] Zoccali M., Giuffrida D., Salafia F., Rigano F., Dugo P., Casale M., Mondello L. (2021). Apocarotenoids profiling in different Capsicum Species. Food Chem..

[18] Caporaso N., Paduano A., Nicoletti G., Sacchi R. (2013). Capsaicinoids, antioxidant activity, and volatile compounds in olive oil flavored with dried chili pepper (*Capsicum annuum*). Eur. J. Lipid Sci. Technol..

[19] Paduano A., Caporaso N., Santini A., Sacchi R. (2014). Microwave and Ultrasound-Assisted Extraction of Capsaicinoids from Chili Peppers (*Capsicum annuum* L.) in Flavored Olive Oil. J. Food Res..

[20] dos Santos Garruti D., de Sousa Mesquita W., Magalhães H.C., da Silva Araújo I.M., de Cassia Alves Pereira R. (2021). Odor-contributing volatile compounds of a new Brazilian tabasco pepper cultivar analyzed by HS-SPME-GC-MS and HS-SPME-GC-O/FID. Food Sci. Technol..

[21] The Good Scent Company. http://www.thegoodscentscompany.com.

[22] Pherobase. http://www.pherobase.com.

[23] Van den Dool H., Kratz P.D. (1963). A generalization of the retention index system including linear temperature programmed gas-liquid partition chromatography. J. Chromatogr. A.

