# Supplementary material for: Elucidation of Analytical–Compositional Fingerprinting of Three Different Species of Chili Pepper by Using Headspace Solid-Phase Microextraction Coupled with Gas Chromatography–Mass Spectrometry Analysis, and Sensory Profile Evaluation
Source: Molecules. 2022 Apr 6;27(7):2355. doi: 10.3390/molecules27072355 (PMC9040714; doi:10.3390/molecules27072355)
Supplement: Supplementary file 1 [file molecules-27-02355-s001.zip › molecules-1631522-supplementary.pdf]

molecules
MDPI

Supplementary material

Elucidation of analytical–compositional fingerprinting of three different species of chili pepper by using Headspace Solid-Phase Microextraction coupled with Gas chromatography–Mass spectrometry analysis, and sensory profile evaluation

Emanuela Trovato [1,*], Federica Vento [2], Donato Creti [3], Paola Dugo [1,2] and Luigi Mondello [1,2,4]

Table S1. Less abundant volatile compounds contained in the chili peppers samples analyzed, expressed in area% as GC-FID measurement results.

	Compound	LRL_{ex}	LRL_{lib}	*Capsicum Chinense*									*Capsicum Annum*					*Capsicum Baccatum*		
				**1**	**2**	**3**	**4**	**5**	**6**	**7**	**8**	**9**	**10**	**11**	**12**	**13**	**14**	**15**	**16**	**17**
15	Tiglic aldehyde	742	738	tr	tr	tr	tr	tr	tr	0.2							tr		0.06	
26	Butanoic acid	791	818		tr		tr	tr		0.17			tr							
32	5-Methyl-3-hexanone	832	842		tr	tr	tr	tr	tr	tr		0.09						tr	tr	tr
34	(*Z*)-2-Hexenal	842	842	tr										0.25	0.14	0.17	0.39	0.42	0.34	0.16
36	Ethyl iso-valerate	850	850		tr	0.17	0.16	tr	0.1			0.24								0.08
45	5-Methyl-3-hexen-2-one	897	894	tr		tr		tr	tr		0.09		0.15	tr	0.13	0.08	tr	tr	tr	tr
46	*n*-Heptanal	902	906	tr	tr	tr	tr	tr	tr		tr	0.14	0.07	tr	0.07	0.06	tr	tr	tr	0.15
47	3-(Methylthio)propionaldehyde	907	909	tr	tr	tr	tr	tr	tr	0.06	0.1	0.14	tr	tr	0.09	0.11	tr	tr	0.05	tr
48	Sorbaldehyde	914	923	tr		tr	tr		tr	tr	0.33	0.44	tr	0.08		0.3	tr	0.36	0.24	0.22
49	Sorbic aldehyde	916	914								0.19	0.48	tr	0.11		0.11	0.46	0.15	0.47	
50	γ-Butyrolactone	918	910	tr	tr	tr		tr		tr		0.18	0.08	0.24		0.12	0.13			0.35
51	3-Methyl-apopinene	921	927				tr		tr			0.07	tr	0.19	0.07		0.38	0.15		0.19
52	Ethyl tiglate	823	933		tr				tr		tr		0.06	tr	tr	tr		tr		0.09
53	α-Thujene	925	927		tr		tr	tr	tr				0.07	0.08	0.09	tr	tr	tr		
54	α-Pinene	931	933				tr					0.13	tr	tr	tr	tr		tr		tr
55	Hexyl formate	936	929				tr			tr	tr	0.06	tr	tr	0.06	0.05	tr	tr		tr
56	4-Methyl-1-hexanol	946	953	tr	tr	tr	tr	tr	tr	tr	tr	0.15	0.05	0.05	0.12	0.05	tr	tr	0.07	tr
57	3-Methyl-cyclohexanone	950	951				tr		tr	0.11	tr	0.08	0.05	tr	tr	tr		tr		tr
58	(*E*)-2-Heptenal	959	956		tr	tr	tr	tr				0.14		tr	tr	0.06	tr	0.06	0.07	0.27

molecules

MDPI

59	Isobutyl butanoate	961	953		tr	tr	tr	tr		0.46	0.06		tr	tr	0.08	0.05	tr	0.09		0.42
61	Ethyl isohexanoate	963	969	0.1	tr	0.2	tr	tr	0.17	0.26	0.05	0.27	0.05	tr	0.12	0.08	tr	0.08	0.05	0.06
62	Benzaldehyde	966	960	tr	tr		tr	tr	tr		tr	0.14	0.06	0.09	0.18	tr	0.09	0.06	tr	tr
63	Isohexanoic acid	968	986	tr	0.07		tr	tr		0.29	0.08		0.08	tr	0.08	tr	tr	0.06	0.06	
64	*n*-Heptanol	970	970	tr	0.15	tr	tr	0.16		tr		0.09	tr	tr		tr		tr	tr	0.07
65	Sabinene	972	972	tr		tr					0.06	0.18		tr	0.1		tr	tr	0.1	tr
66	β-Pinene	975	978			tr					tr	0.37	tr	tr	0.08			tr		tr
67	2-Ethylbutyl acetate	976	972	tr		tr	tr			tr	0.09				0.44	0.16	tr	tr		0.14
68	3-Methylmercapto-1-propanol	985	982	tr			tr		0.05					0.06	0.39	0.36				
72	Isobutyl 2-methylbutanoate	1001	1002	tr	tr	tr	tr	tr	0.05	tr	tr	0.18	0.07		0.11	tr		tr	0.07	tr
73	*n*-Octanal	1002	1006	tr		tr	tr	tr	tr	tr	tr	0.21	tr	tr	0.38	0.06	tr	tr	0.05	tr
74	*p*-Mentha-1(7),8-diene	1003	1004	tr	tr	tr	tr	tr		tr	tr			tr			tr	0.05	0.07	0.33

molecules

MDPI

	Compound	LRL_{ex}	LRL_{lib}	*Capsicum Chinense*									*Capsicum Annum*					*Capsicum Baccatum*		
				1	2	3	4	5	6	7	8	9	10	11	12	13	14	15	16	17
75	Isobutyl isovalerate	1005	1005	0.06	tr	0.07	tr	tr	0.18	0.14	tr			0.06		0.15		0.13	tr	0.22
76	Ethyl (*Z*)-hex-3-enoate	1006	1003		tr	tr	tr	0	tr		tr	0.14		tr		0.06	0.08		tr	0.43
77	Isopentyl- isobutanoate	1011	1014		tr	tr	tr	tr	tr	tr	tr	0.1	tr	0.05	tr	tr			tr	
78	Pentyl 2-methylpropanoate	1014	1016	tr	tr	tr	tr	0.23	tr	0.12	tr	0.17		tr		tr	0.08	tr	0.09	0.15
79	(*E*)-6-Methylhept-4-en-1-ol	1020	1019	0.08	tr	0.23	0.13		tr	0.29		0.06	tr	tr	0.15	0.06	0.05	tr	0.09	tr
82	2-Ethyl-1-hexanol	1029	1031	tr	0.05		tr	tr	tr	tr	0.07		0.1	0.21	0.44	0.43	tr	0.09	0.1	0.26
83	3-Methyl-1-heptanol	1034	1041	tr	tr	tr	tr	tr			0.16	0.24					tr	tr	tr	tr
85	Benzyl alcohol	1036	1040	tr		tr	tr	tr	tr	tr	0.1	0.14	tr	0.1	0.11	0.15	tr	tr	0.1	tr
86	9-Methyl-1-decene	1038	1041	tr	tr	tr	tr		tr	tr	tr	0.32	0.06	0.05		0.22	tr	0.14	0.13	tr
87	Phenylacetaldehyde	1043	1045	0.12	tr	tr	tr	tr	tr	0.05	0.07	0.14	0.07	0.16	0.17	0.19	0.28	tr	0.06	0.19
88	5-Methyldecane	1044	1054					tr	tr		tr				0.17	tr	0.09		0.27	
89	Amyl pivalate	1045	1047	tr		tr	0.1	0.07	0.06		tr		0.06	0.34				0.06		
92	2-Methyloctanal	1052	1059	tr	tr	tr			tr	0.11	tr	0.14	0.22	tr	0.06	tr	tr	0.28	tr	0.05
93	γ-Terpinene	1055	1058	tr			tr	tr	tr	tr	0.19	0.18	0.18	0.06	0.17	0.06	tr	0.19		0.4
95	*n*-Octanol	1071	1076	tr	tr	tr	tr	0.11	tr	0.12		0.08	tr	0.05	0.23	0.16	tr	tr	0.06	tr
96	(*E*)-3-Octen-1ol	1074	1081	tr	tr	tr	tr			tr	tr	0.13	0.06	tr	0.22	0.05	tr	0.05	0.06	0.13
97	*n*-Heptanoic acid	1080	1116	tr	tr	tr	0.07		tr	tr		0.1	tr	0.05	0.12	0.05	0.1	0.16	0.12	0.06
102	2-Methylbutyl 2-methylbutanoate	1101	1104	tr	tr	tr	tr	tr	0.07	tr	tr			tr		tr	tr	0.26	tr	0.11
108	Phenethyl alcohol	1113	1113	0.06		tr	tr	tr	tr	tr	tr	0.16	tr	tr	tr	tr	tr	tr	0.3	tr
109	*p*-Menta-1,3,8-triene	1122	1112	tr		tr				tr	tr	tr		tr	tr	tr	tr	tr		0.42
110	2-Ethylhexoic acid	1127	1137	tr		0.19			0.13	tr	tr	0.07		tr	tr	tr	tr	0.12		0.28
113	Pentyl 2-methylbutanoate	1136	1142	tr	tr	tr	0.19	tr	0.29	tr	0.19	0.15	0.08	tr	0.36	0.11	0.07		0.21	tr
115	(*Z*)-3-Hexenyl isobutanoate	1141	1146	tr	0.06	tr	0.1		0.1	0.11	tr	0.1				0.09		tr		
118	3-Methylbut-2-enyl 3-methylbutanoate	1148	1150	tr		0.1	tr	0.06	0.16		tr	0.06			tr	tr				
119	Dictyopteren D'	1150		0.21	0.16	0.1	0.22		0.16		tr	0.08			0.09	tr	0.29			
120	neo-Isopulegol	1152	1148	tr	0.1	0.05	tr	tr	tr	0.07	tr	0.18	0.21	0.05	0.09	0.05	tr	0.05	0.06	0.08
121	(*E*)-Pinocamphone	1160	1160	tr	tr	tr	tr			tr	tr	0.15	tr	0.06	0.1	0.11	tr	tr		0.36
122	Tetrahydrolavandulol	1168	1162			tr		tr	tr	0.22		0.28	tr	tr	0.11	0.13	0.05	tr	0.05	
123	Ethyl benzoate	1170	1170	tr	tr	tr	tr	tr		tr	tr	0.23		tr	0.13	tr		tr		0.22
124	Benzenecarboxylic acid	1175	1213	tr	tr	tr	tr			tr		0.1	0.1	tr						tr

molecules

MDPI

126	Neoisomenthol	1182	1189	tr			tr		0.1				tr							0.1
127	Terpinen-4-ol	1183	1184					tr		tr		0.32								
128	Nonanol	1185	1176	tr	tr	0.12	0.28	tr	0.24	tr	tr	0.12		tr					0.06	

	**Compound**	LRL_{ex}	LRL_{lib}	*Capsicum Chinense*									*Capsicum Annum*					*Capsicum Baccatum*		
				**1**	**2**	**3**	**4**	**5**	**6**	**7**	**8**	**9**	**10**	**11**	**12**	**13**	**14**	**15**	**16**	**17**
129	*n*-Octanoic acid	1188	1192	0.07	tr	tr	tr	tr	tr	tr		0.06	tr	tr	0.12	0.09			tr	0.06
130	Ethyl (4Z)-4-octenoate	1191	1184	tr			tr	tr	tr	0.06	tr				0.06	tr	tr	tr		tr
131	Hexyl butanoate	1192	1195	tr	tr	tr			0.07	tr	tr	0.07	tr		0.1	0.06	tr	tr	tr	tr
132	Methyl salicylate	1193	1192	tr	tr	tr	0.05	tr		tr	0.11	0.33	tr	0.29	0.12	tr	tr	0.05	0.07	tr
133	*n*-Dodecane	1196	1187		tr	tr							tr	tr	0.12	0.07	tr	tr	0.05	0.15
136	Pelargol	1208	1200	tr			tr	tr	tr	tr	tr	0.23	tr	0.06	0.07	0.13		tr	tr	tr
137	4-Methylhexyl isobutanoate	1215	1220	0.09	0.05	tr	0.32	tr	tr	tr	tr	0.08		tr	0.08	0.06	tr	tr	0.06	
139	ESTER	1226		0.4	0.29	0.34	0.1	0.14	0.18	0.17	0.4	0.11	0.06	tr	0.09	0.05	0.06	tr	tr	tr
146	ESTER	1257		0.06	0.05	tr	tr	tr	tr	tr	tr			tr	0.14	0.07	tr	tr	tr	tr
147	2-Methyldodecane	1263	1265	tr	tr	tr	0.07	tr	0.1	tr	tr	0.1	tr	tr	0.17	tr	0.06	tr	0.05	tr
148	Ethyl salicylate	1273	1270	0.18	tr	0.13	tr	tr	0.12	0.09	0.05	0.18	tr	tr	0.08	0.05	tr	tr	0.07	0.37
149	*n*-Nonanoic acid	1275	1289	0.09	tr	0.08	0.13	0.11	0.08	tr	0.09	0.14	0.06	tr	0.19	0.06		tr	tr	tr
150	Hexyl angelate	1285	1283	0.19	tr	tr	0.26		tr	tr	tr	0.07	tr	tr	0.18	tr	tr	tr	tr	tr
153	5-Methylhexyl 2-methylbutanoate	1294	1299	tr	0.08	tr	0.23	tr	tr	tr				tr		0.1	tr			
154	*n*-Tridecane	1298	1300	0.06	tr	tr	0.08		tr	tr	tr	0.2	tr	0.14	0.19		0.1	tr	0.06	0.09
156	Hexyl pentanoate	1301	1293		0.12		0.11	0.29							0.1	tr				
161	Dihydro citronellol acetate	1316	1319		tr	tr	tr	0.07		tr		0.1	tr	tr	tr	tr	tr	tr	tr	0.09
162	(E)-3-Hexenyl tiglate	1317	1319	0.09	0.19	0.08	0.1	tr	tr	tr	tr	0.19		tr		tr	tr	tr	0.06	tr
163	(Z)-3-Hexenyl tiglate	1321	1325	0.11	0.07	0.06	tr	0.07	0.11	tr	0.25	0.1	tr	tr	0.09	tr	tr			tr
164	Hexyl tiglate	1328	1329	0.06	0.16	0.07	0.07	0.26	0.1	tr	tr	0.08				tr	tr			tr
166	1-Nonadecyne	1334		0.05			0.16		0.07	tr		0.18				tr			tr	
168	Octyl isobutanoate	1342	1346	tr	tr	0.08	0.18	0.11	tr			0.06				tr		tr	tr	
169	(*Z*)-Hept-3-enyl 2-methylbutanoate	1345		0.09	0.28	0.09	0.05	0.08	0.1	0.06		0.07			0.11	tr				
173	Ethyl 8-methylnonanoate	1358	1359	0.15	0.2	0.18	0.28	0.05	tr	0.06	tr	0.07		tr	0.06	tr	0.1		tr	tr
175	*n*-Decanoic acid	1374	1398	tr	tr	0.33	tr	tr	tr	tr	tr			tr	0.09		tr		tr	tr
184	Benzyl isovalerate	1395	1399		0.14		0.15	tr	0.06	tr	tr									
185	*n*-Tetradecane	1397	1400	tr		tr			0.09	tr	0.06	0.24	0.05	0.05	0.23	0.1	tr	tr	0.08	0.06
187	*n*-Dodecanal	1409	1412	tr	0.06	tr	tr	tr	tr	tr	tr	0.06	tr	tr	0.13	0.05	tr	tr	0.07	tr

188	(Z)-α-Bergamotene	1411	1416	tr	tr	tr	tr	tr	tr		tr	0.09		tr	0.13	0.13	tr	tr	tr	tr
189	Longifolene	1413	1412	tr	0.1	tr	tr	tr	tr	tr		0.18				tr	tr	tr	0.09	
190	α-Cedrene	1416	1414	0.12	tr	tr	0.2	0.29	tr	tr	0.09		tr			0.1	0.07			
191	(E)-Caryophyllene	1420	1424		0.1	tr	tr	tr	tr	tr	tr					tr	0.12	0.32		tr
193	γ-Elemene	1427	1432	0.09	0.26	0.12	0.12	0.16	0.08	tr		0.12					0.1			
							Capsicum Chinense								*Capsicum Annum*				*Capsicum Baccatum*	
	**Compound**	LRL_{ex}	LRL_{lib}	**1**	**2**	**3**	**4**	**5**	**6**	**7**	**8**	**9**	**10**	**11**	**12**	**13**	**14**	**15**	**16**	**17**
194	β-Copaene	1431	1433	0.3	0.29	0.16	0.08	0.15	tr	tr		0.24	tr		tr		0.2		tr	
195	Octyl 2-methylbutanoate	1434	1433	0.07	0.39	0.19	0.14	0.25	0.07		tr				0.31	0.13				
201	Cadina-3,5-diene	1449	1452	tr	tr		0.07	0.12				0.1		tr	0.13					
203	(*E*)-β-Farnesene	1453	1452				tr	0.28		tr				tr	0.36	0.09	tr	tr	0.21	
204	α-Humulene	1456	1454	0.07	0.13	0.08	0.06	0.34	0.06	tr	tr			tr	0.1		tr	tr	tr	
205	α-Patchoulene	1461	1459	0.08			0.13	0.11	tr	tr			0.08		tr	tr			tr	tr
208	Cadina-1(6),4-diene	1474	1474	0.1	0.1	0.09	0.13	0.07	0.12	tr	tr	0.2				tr	tr		0.1	tr
209	γ-Muurolene	1475	1478	0.16	0.27	0.08	0.2	0.1	0.11	tr	tr	0.32		tr	tr		tr	tr	0.28	tr
215	6-Methylhept-4-en-1-yl 2-methylpentanoate	1493	1484	0.14	0.22	0.25	0.13	0.24	tr	tr		tr		0.07	tr	tr	0.1	tr	0.27	0.05
216	γ-Amorphene	1494	1490	0.18	0.15	0.08	0.12	0.08	tr	tr		0.09					tr		0.07	
221	ESTER	1512		tr	tr	0.08	tr	tr	tr						0.1	tr	tr			tr
222	γ-Cadinene	1514	1512	0.16	0.14	0.05	0.11	0.08	tr	tr					0.08	tr		tr	tr	tr
224	(*E*)-Calamenene	1522	1527	0.36	0.2	0.22	0.36	0.16	0.16	tr	tr	0.13			0.08	0.06		tr	0.09	tr
225	Citronellyl butanoate	1533	1529	0.09	0.06	0.1	0.06	0.05	0.07	tr		0.07				tr	tr			tr
226	(*E*)-Cadina-1,4-diene	1534	1536	tr		0.09	tr	0.08	tr		tr	0.11		tr	0.15	tr	0.16		tr	tr
228	ESTER	1546		0.13	0.14	0.16	0.06	0.06	tr	tr	tr	0.08			0.09	tr	tr			tr
229	Dodecanethiol	1547	1543	tr	tr	0.08	0.09	tr	tr	tr	tr	0.07		tr	0.16	tr	tr			
230	Geranyl butanoate	1551	1559	0.06	0.17	tr	0.07	0.2								tr				
231	(*E*)-Nerolidol	1558	1561			tr											0.4			
232	12-Methyl-oxa-cyclododecan-2-one	1559		0.06			0.12	tr							0.06	tr				
233	2-Methyl pentadecane	1561	1567	0.44	0.18	0.14	0.19	0.06	tr	tr	tr	0.17		0.1	0.12	tr	tr	tr	tr	tr
234	3-Methyl pentadecane	1568	1574	0.08	0.05	tr	tr	tr	tr	tr	tr	0.08		tr	0.12	tr	tr	tr		tr
236	(*Z*)-2-Tridecen-1-ol	1578	1572	tr	tr	0.13	tr	0.06	tr				tr							tr
239	Ethyl dodecanoate	1591	1598	0.19	0.12		0.14	0.08	0.07				tr		0.07	tr	tr			tr

molecules

MDPI

240	*n*-Hexadecane	1598	1600	tr	0.05	0.06	tr	tr	0.07	tr	tr	0.15		0.06	0.11	tr	0.07	tr		tr
241	8-Methylnonanoic acid, 2-methylbutyl ester	1606	1610	0.42	0.35	0.2	0.16	0.08	tr	tr						0.07	0.21			
242	α-Corocalene	1543	1544	tr	tr	tr	tr	tr	tr		tr				0.11	tr				
243	Isopentyl 8-methylnon-6-enoate	1594	1592	tr	tr	0.14	tr	tr	tr	tr						tr	tr			tr
244	Oxacyclotetradecan-2-one	1629	1632	0.08	0.25	0.36	0.35	tr	tr	tr		0.15			0.15	0.06	0.21		tr	
245	14-Methyl-oxacyclotetradecan-2-one	1650	1660	0.15	0.05	0.12	0.27	tr		tr	tr				0.12	tr	0.4			tr
	**Compound**	LRL_{ex}	LRL_{lib}	***Capsicum Chinense***									***Capsicum Annum***					***Capsicum Baccatum***		
				**1**	**2**	**3**	**4**	**5**	**6**	**7**	**8**	**9**	**10**	**11**	**12**	**13**	**14**	**15**	**16**	**17**
246	Cadin-4-en-10-ol	1657	1659	0.11	tr		tr	0.12	tr	tr					0.17	tr		tr		tr
247	2-Methyl hexadecane	1660	1664	0.32	0.11	0.15	0.08	tr	tr	tr		0.16			0.23	tr	0.23			
248	8-hydroxy-isobornyl isobutanoate	1665	1676	tr	0.06	0.08	0.06	tr	tr	tr	tr	0.12	tr	0.07	0.18	tr		tr	tr	0.05
251	*n*-Heptadecane	1696	1700	0.35	0.22	0.18	0.1	tr	0.06	tr	tr	0.2		0.07	0.1	tr	tr	tr		tr
253	Hexyl 8-methylnon-6-enoate	1720	1730	tr	tr	0.28	tr	tr	tr	tr	tr	0.11		tr	0.12	tr	tr	tr		
254	2-Methyl-heptadecane	1762	1763	tr	tr	tr	tr	tr	tr			0.05						tr		
255	(*Z*)-2-Pentadecenal	1764	1760	tr	tr	tr	tr	tr	tr						0.1					
256	3-Methyl-heptadecane	1767	1774	tr	tr	tr	tr	tr	tr	tr					0.09		tr			
257	(*Z*)-2-Pentadecenol	1778	1776	tr	tr	tr	tr	tr	tr	tr	tr	0.12	tr		0.1				tr	tr
258	1-Tetradecyl acetate	1804	1810	tr	tr	tr	tr	tr	tr	tr	tr				0.14					
259	Pentadecylic acid	1823	1869		tr	tr		0.05		tr					0.16					
260	Pentadecanolide	1830	1827	tr	tr	tr	tr	tr							0.16					tr
263	ESTER	1861		tr	tr	0.24	tr	tr	tr											
263	*n*-Hexadecanol	1884	1884	tr	tr	tr	tr	0.08	tr											
264	n-Heptadecanal	1915	1918	tr	tr	tr	tr	tr	tr	tr		0.07	tr		0.08		tr			tr
265	Hexadecanolact-16-one	1934	1938	tr	tr	tr	tr	tr	tr				tr		0.14	tr				tr
266	1-Hexadecanol acetate	2003	2009	tr	tr	tr	tr	tr				0.08					tr			
267	Octadecyl acetate	2205	2212	tr	tr	tr		tr		tr									0.07	
268	*n*-Heptacosane	2699	2700					tr										0.05		268
269	*n*-Octacosane	2798	2800					tr				0.27					tr	tr		269
	**Total**			**6.63**	**6.44**	**6.58**	**7.19**	**5.16**	**3.81**	**3.22**	**2.88**	**14.00**	**2.18**	**3.30**	**12.3**	**5.42**	**5.13**	**3.43**	**5.21**	**6.91**

The compounds number is reported in order of elution, considering the total number of compounds eluted. For the identification of the compounds not reported in this table, see Table 1. tr = trace compound.

Table S2. Volatile compounds contained in the chili-pepper-flavored olive oil samples analyzed, expressed in area% as GC-FID measurement results.

	Compound	LRI_{exp}	LRI_{lib}	EVO1	EVO2	EVO3
1	(E)-2-Butenal	619	629	0.08	0.09	0.03
4	Acetic acid	659	661	1.84	1.59	1.39
9	3-Penten-2-one	690	691		1.53	
11	Acetoin	726	716	0.04		0.04
13	Isopentyl alcohol	733	729	0.03	0.04	0.05
14	sec-Butylcarbinol	738	733			0.04
19	Toluene	764	763	0.12		
24	3-Methylcrotonaldehyde	787	780	0.01		0.03
28	n-Hexanal	801	801	5.07	5.39	1.70
33	4-Methyl-1-pentanol	838	832		0.03	0.60
34	(Z)-2-Hexenal	842	842	0.27	37.41	0.21
37	(E)-2-Hexenal	850	850	30.38	2.01	19.21
41	(E)-2-Hexenol	864	864	6.33	2.39	12.64
42	n-Hexanol	868	867	4.30	2.95	3.34
44	n-Pentanoic acid	889	918	0.04		0.08
46	n-Heptanal	902	906	0.65	0.29	0.11
48	Sorbaldehyde	914	923	0.12	0.38	0.04
49	Sorbic aldehyde	916	914	0.20	0.20	0.03
53	α-Thujene	925	927			0.02
54	α-Pinene	931	933	0.05	0.01	0.02
58	(E)-2-Heptenal	959	956	1.34	0.78	0.51
62	Benzaldehyde	966	960	0.18	0.20	0.18
64	n-Heptanol	970	970	0.09		0.06
65	Sabinene	972	972	0.06	0.04	0.08
66	β-Pinene	975	978	0.13		0.14
69	6-Methyl-hept-5-en-2-one	984	986	1.12	0.30	0.18
80	p-Cymene	1025	1024	0.29	0.10	0.04
81	Limonene	1028	1030	1.82	0.46	0.22
84	(Z-β-Ocimene	1035	1035			0.13
85	Benzyl alcohol	1036	1040		0.22	0.26
90	(E)-β-Ocimene	1046	1046	1.23	0.49	1.16
94	(E)-2-Octenal	1067	1058	0.13	0.11	0.05
95	n-Octanol	1071	1076	0.09	0.08	0.02
98	Guaiacol	1086	1094	0.03	0.16	
100	3-Methylbutyl 2-methylbutyrate	1098	1104			0.18

103	n-Nonanal	1103	1107	5.30	2.11	1.46
105	2-Methylbutyl isovalerate	1106	1109			0.07
111	(4E,6Z)-Alloocimene	1128	1128	0.07	0.05	0.05
113	Pentyl 2-methylbutyrate	1136	1142			0.2
114	(E,E)-Allocimene	1140	1145	0.08		
115	(Z)-3-Hexenyl isobutyrate	1141	1146			0.32
116	Pentyl isovalerate	1142	1143			0.63
117	Hexyl isobutyrate	1146	1150		0.02	0.27
123	Ethyl benzoate	1170	1170			0.06
127	Terpinen-4-ol	1183	1184	0.03		
132	Methyl salicylate	1193	1192	0.24	0.10	0.16
133	*n*-Dodecane	1196	1200	0.03		0.04
134	4-Methylpentyl 2-methylbutanoate	1198	1202		0.42	3.85
135	4-Methylpentyl 3-methylbutanoate	1209	1206			11.12
136	Pelargol	1208	1200	1.46	1.66	
137	4-Methylhexyl isobutyrate	1215	1220			0.02
141	(Z)-3-Hexenyl 2-methylbutyrate	1233	1231		0.11	
143	Hexyl 3-methylbutyrate	1246	1243		0.05	1.97
144	(E)-Hex-2-enyl 3-methylbutanoate	1248	1243	0.03		0.29
151	6-Methylhept-4-en-1-yl isobutyrate	1289	1293			0.11
152	3-Methylpentyl (2E)-2-methyl-2-butenoate	1291	1300			0.06
153	5-Methylhexyl 2-methylbutanoate	1294	1299			0.03
155	5-Methylhexyl 3-methylbutanoate	1300	1303			0.14
159	4-Methylhexyl 2-methylbutanoate	1308	1307			0.03
160	4-Methylpentyl 4-methylpentanoate	1313	1315			0.28
163	(Z)-3-Hexenyl tiglate	1321	1325			0.03
165	Heptyl 2-methylbutyrate	1332	1333			0.04
167	Heptyl isovalerate	1338	1338			0.17
168	Octyl isobutyrate	1342	1346			0.03
170	α-Cubebene	1347	1347		0.03	0.11
177	Cyclosativene	1370	1367	0.23	0.09	0.61
178	α-Copaene	1376	1375	1.28	0.44	3.00
180	6-Methylhept-4-en-1-yl 3-methylbutanoate	1385	1388	0.01		0.48
181	β-Elemene	1389	1390	0.02		0.02
183	6-Methylheptyl 2-methylbutanoate	1394	1398			0.06
184	Benzyl isovalerate	1395	1399			0.06
185	n-Tetradecane	1397	1400	0.02	0.04	0.07
188	(Z)-α-Bergamotene	1411	1412	0.01		
191	(E)-Caryophyllene	1420	1424	0.05	0.02	
194	β-Copaene	1431	1433			0.06
196	(E)-α-Bergamotene	1435	1432		0.08	
203	(E)-β-Farnesene	1453	1452		0.01	0.01

204	α-Humulene	1456	1454			0.01
208	Cadina-1(6),4-diene	1474	1474			0.01
211	γ-Himachalene	1483	1481		0.12	0.37
223	δ-Cadinene	1519	1512	0.03	0.02	0.05
224	(E)-Calamenene	1522	1527			0.03
	**Total**			**64.93**	**62.62**	**69.17**

The compound's number is reported in order of elution, considering the total number of compounds identified in chili peppers.

Table S3. Standard key compounds used for the training of panelists for sensorial analysis.

	Compound	
1	Isovaleric aldehyde	590-86-3
2	2-Methylbutyraldehyde	96-17-3
3	3-Methyl-2-butanol	598-75-4
4	Acetoin	513-86-0
5	Isopentyl alcohol	123-51-3
6	Isobutyric acid	79-31-2
7	2,3-Butadienol	19132-06-0
8	Isopentyl formate	110-45-2
9	Butanoic acid	107-92-6
10	(*Z*)-3-Hexenal	6789-80-6
11	*n*-Hexanal	66-25-1
12	Ethyl 2-methylbutyrate	53956-13-1
13	(*E*)-2-Hexenal	505-57-7
14	Isovaleric acid	503-74-2
15	*n*-Hexanol	111-27-3
16	2-Methylbutyric acid	116-53-0
17	*n*-Pentanoic acid	109-52-4
18	3-(Methylthio)propionaldehyde	3268-49-3
19	4-methylpentanoic acid	646-07-1
20	3-Methylmercapto-1-propanol	505-10-2
21	*n*-Hexanoic acid	142-62-1
22	Ethyl hexanoate	123-66-0
23	Isobutyl 2-methylbutyrate	2445-67-2
24	Phenylacetaldehyde	122-78-1
25	Guaiacol	90-05-1
26	2-Methylbutyl 2-methylbutyrate	2445-78-5
27	3-methylbutyl 3-isovalerate	659-70-1
28	2-Methylbutyl isovalerate	2445-77-4
29	3-Methylpentyl isobutyrate	84254-84-2
30	Phenethyl alcohol	60-12-8
31	(*Z*)-3-Hexenyl isobutyrate	41519-23-7
32	Pentyl isovalerate	25415-62-7
33	3-Methoxy-2-isobutylpyrazine	24683-00-9
34	Methyl salicylate	119-36-8
35	4-Methylpentyl 2-methylbutanoate	35852-40-5
36	4-Methylpentyl 3-methylbutanoate	850309-45-4
37	(*Z*)-3-Hexenyl 2-methylbutyrate	53398-85-9
38	(*Z*)-3-Hexenyl 3-methylbutyrate	35154-45-1
39	Hexyl 3-methylbutyrate	10032-13-0
40	(E)-Hex-2-enyl 3-methylbutanoate	68698-59-9

41	Heptyl isobutyrate	2349-13-5
42	Ethyl salicylate	118-61-6
43	*n*-Nonanoic acid	112-05-0
44	4-Methylhexyl 2-methylbutanoate	850309-46-5
45	4-Methylpentyl 4-methylpentanoate	35852-42-7
46	*n*-Decanoic acid	334-48-5
47	(E)-Caryophyllene	87-44-5

Table S4. Contribution of the variables on PC1 and PC2.

	Compound	Without EVOOs PC1	Without EVOOs PC2	With EVOOs PC1	With EVOOs PC2
1	(E)-2-Butenal	0.000	0.034	0.000	0.045
2	3-methyl-2-Butanone	0.001	0.110	0.000	0.144
3	Isovaleric aldehyde	0.001	0.128	0.000	0.145
4	Ethanoic acid	0.024	73.790	0.121	69.630
6	2-Methylbutyraldehyde	0.007	0.288	0.003	0.296
7	3-Methyl-2-butanol	0.005	0.039	0.003	0.043
8	(Z)-2-Buten-1-ol	0.039	0.000	0.026	0.002
9	3-Pentenone	0.013	0.062	0.002	0.031
10	Propionic acid	0.008	0.027	0.005	0.034
11	Acetoin	0.174	23.219	0.269	22.832
12	Isoprenol	0.017	0.097	0.011	0.104
13	Isopentyl alcohol	0.005	0.067	0.002	0.086
14	sec-Butylcarbinol	0.001	0.158	0.002	0.163
16	Isopropyl ethyl ketone	0.003	0.002	0.002	0.003
17	Ethyl iso-butyrate	0.007	0.030	0.004	0.038
18	Isobutyric acid	0.001	0.038	0.001	0.039
19	Pentyl alcohol	0.000	0.001	0.000	0.000
20	Toluene	0.027	0.000	0.021	0.001
21	(E)-2-Penten-1-ol	0.001	0.006	0.001	0.009
25	2,3-Butadienol	0.024	0.000	0.017	0.002
27	(Z)-3-Hexenal	0.002	0.004	0.002	0.006
28	n-Hexanal	13.869	0.129	10.528	0.180
29	Ethyl lactate	0.029	0.001	0.020	0.003
30	2-Hexanol	0.127	0.001	0.089	0.009
31	4-Methyl-2-pentenal	0.199	0.000	0.142	0.001
34	Isovaleric acid	0.000	0.030	0.000	0.038
35	4-Methyl-1-pentanol	0.472	1.281	0.460	0.358
36	(Z)-2-Hexenal	0.007	0.000	0.782	5.406
37	Ethyl 2-methylbutyrate	0.007	0.006	0.007	0.001
38	(E)-2-Hexenal	83.586	0.162	85.784	0.226
40	(E)-3-Hexenol	0.080	0.027	0.045	0.000
41	(Z)-3-Hexenol	0.008	0.010	0.008	0.003
42	(E)-2-Hexenol	0.213	0.170	0.596	0.000

43	n-Hexanol	0.734	0.009	0.743	0.058
58	(E)-2-Heptenal	0.000	0.000	0.003	0.007
68	6-Methyl-hept-5-en-2-one	0.000	0.015	0.002	0.008
81	Limonene	0.000	0.002	0.003	0.000
90	(E)-β-Ocimene	0.288	0.013	0.247	0.010
91	Isopentyl butyrate	0.000	0.009	0.000	0.010
99	Isobutyl tiglate	0.001	0.008	0.000	0.009
103	n-Nonanal	0.000	0.014	0.033	0.014
106	3-Methylpentyl isobutyrate	0.007	0.001	0.007	0.000
114	(E,E)-Allocimene	0.005	0.001	0.004	0.000
116	Pentyl isovalerate	0.007	0.009	0.005	0.007

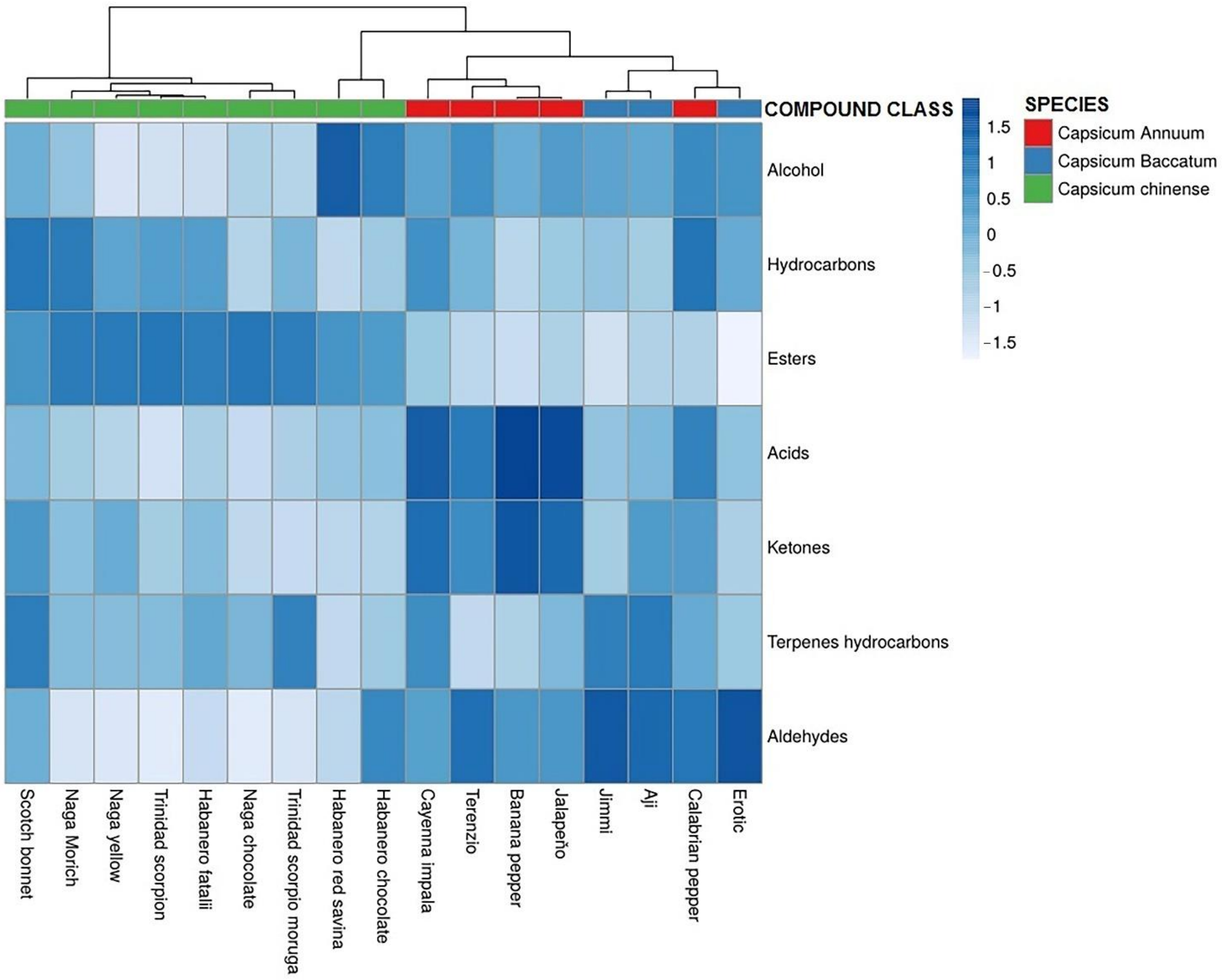

Figure S1. Hierarchical cluster analysis based on relative percentage areas of identified compounds classes. Original values are ln(x + 1)-transformed. Rows are centered; Pareto scaling is applied to rows. Imputation is used for missing value estimation. Columns are clustered using correlation distance and average linkage. There is a total of 7 rows and 17 columns.

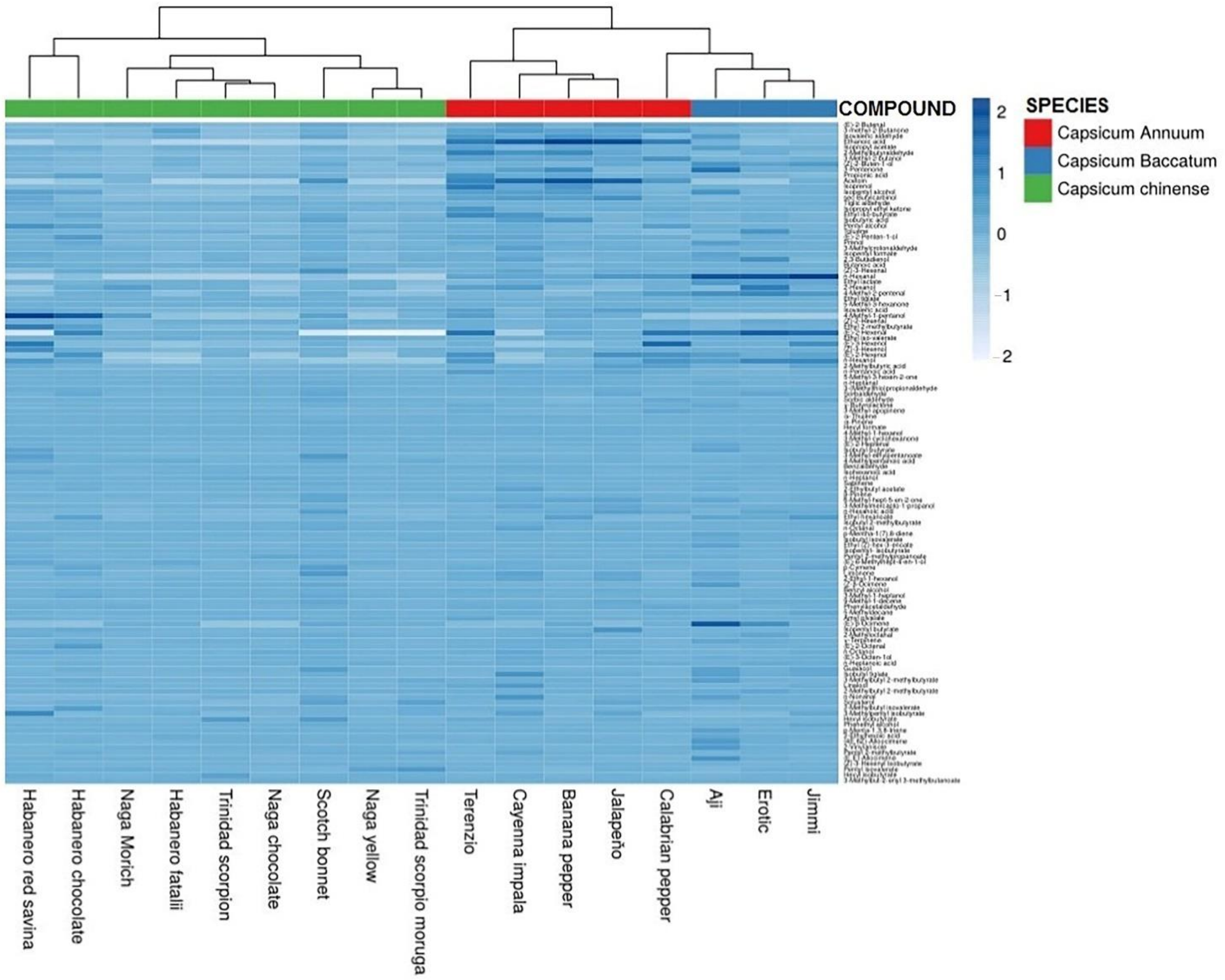

Figure S2. Hierarchical cluster analysis based on relative percentage areas of the 118 most abundant identified volatiles. Original values are ln(x + 1)-transformed. Rows are centered; Pareto scaling is applied to rows. Imputation is used for missing value estimation. Columns are clustered using correlation distance and average linkage. There is a total of 118 rows and 17 columns.

Capsicum chinense

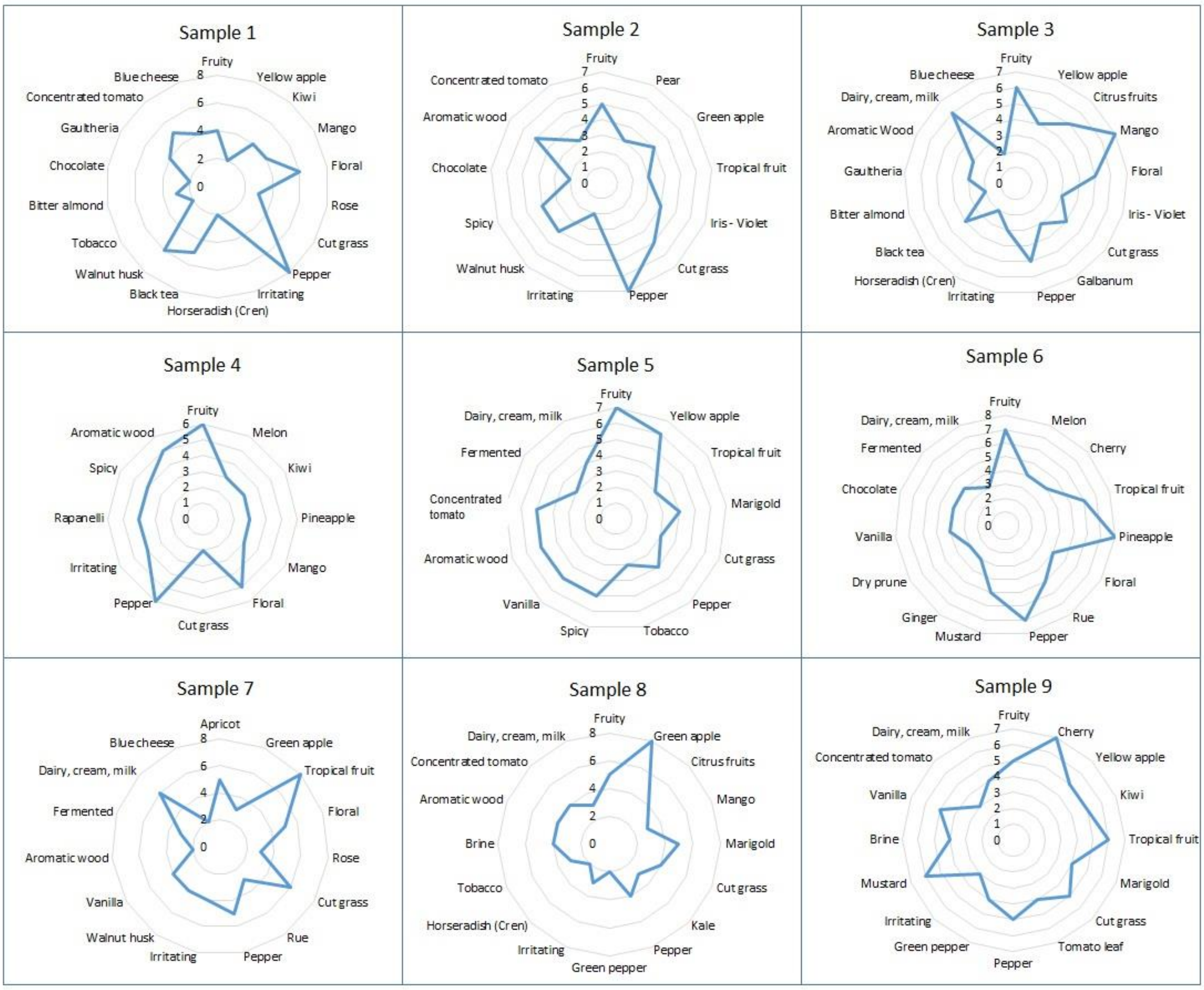

Figure S3. Aroma profile of the *Capsicum chinense* pepper from descriptive sensory analysis on line scale (n = 10). Sample 1 (Naga Morich), sample 2 (Trinidad Scorpion), sample 3 (Habanero Fatalii), sample 4 (Naga Yellow), sample 5 (Naga Chocolate), sample 6 (Trinidad Scorpio Moruga), sample 7 (Habanero Red Savina), sample 8 (Habanero Chocolate), and sample 9 (Scotch Bonnet).

Capsicum annuum

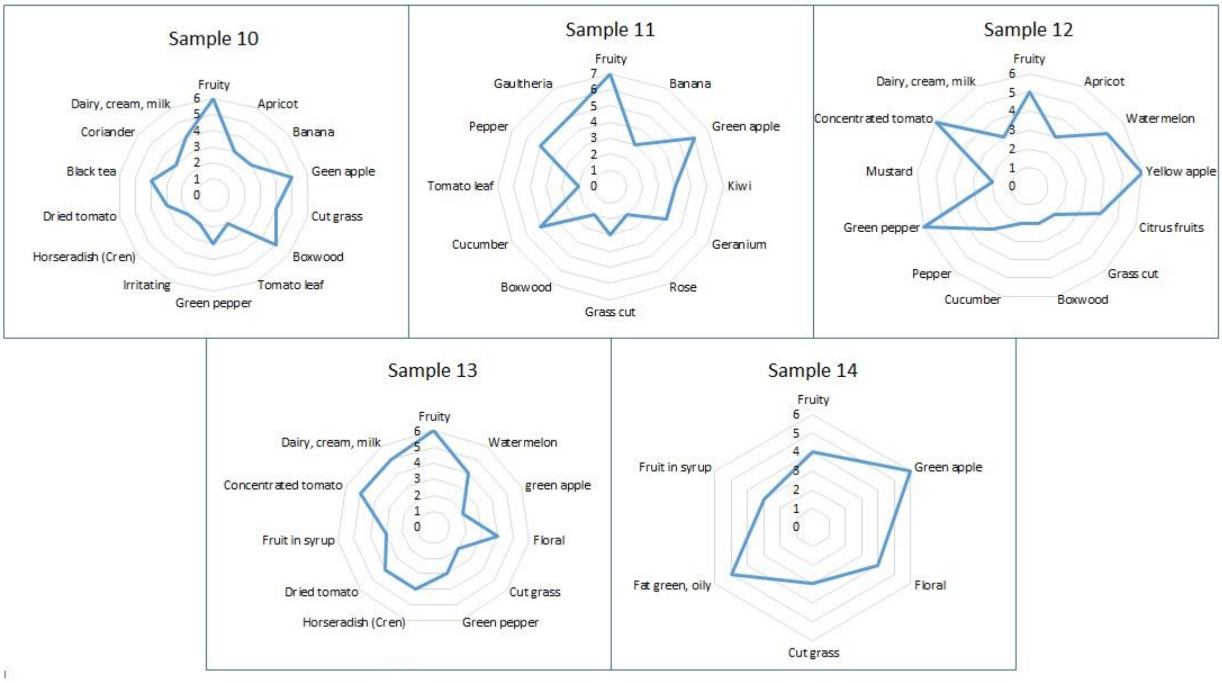

Figure S4. Aroma profile of the *Capsicum annuum* pepper from descriptive sensory analysis on line scale (n = 10). Sample 10 (Banana Pepper), sample 11 (Terenzio), sample 12 (Cayenna Impala), sample 13 (Jalapeňo), and sample 14 (Calabrian pepper).

Capsicum baccatum

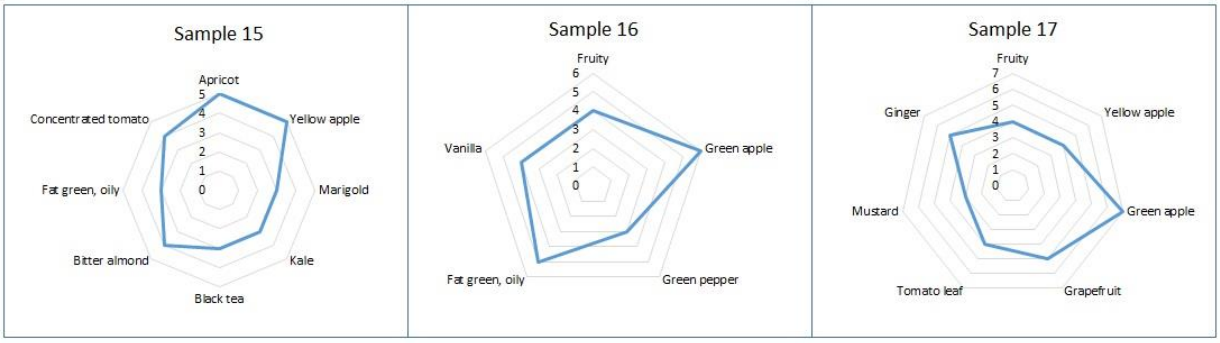

Figure S5. Aroma profile of the *Capsicum baccatum* pepper from descriptive sensory analysis on line scale (n = 10). Sample 15 (Erotic), sample 16 (Jimmi), and sample 17 (Aji limòn).

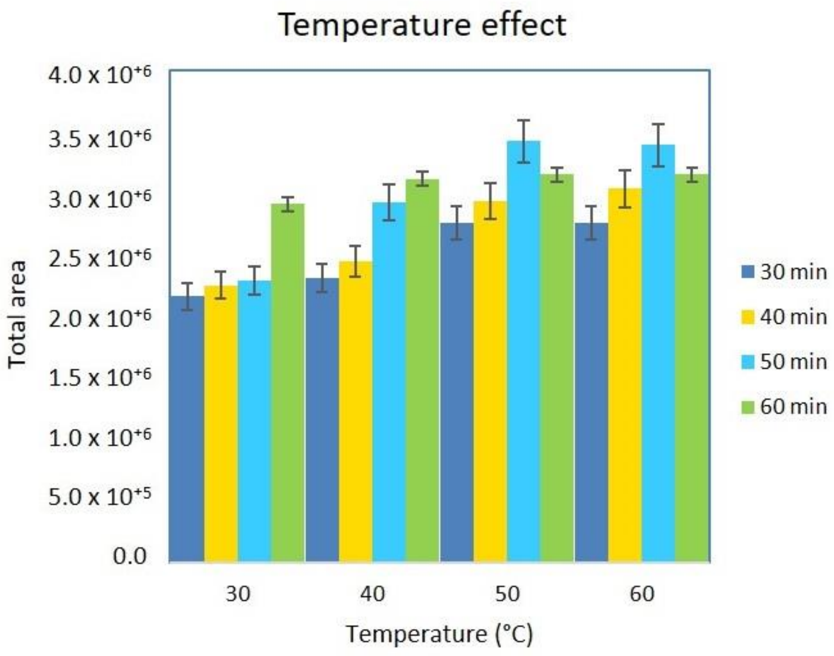

Figure S6. Influence of temperature on SPME method extraction optimization.

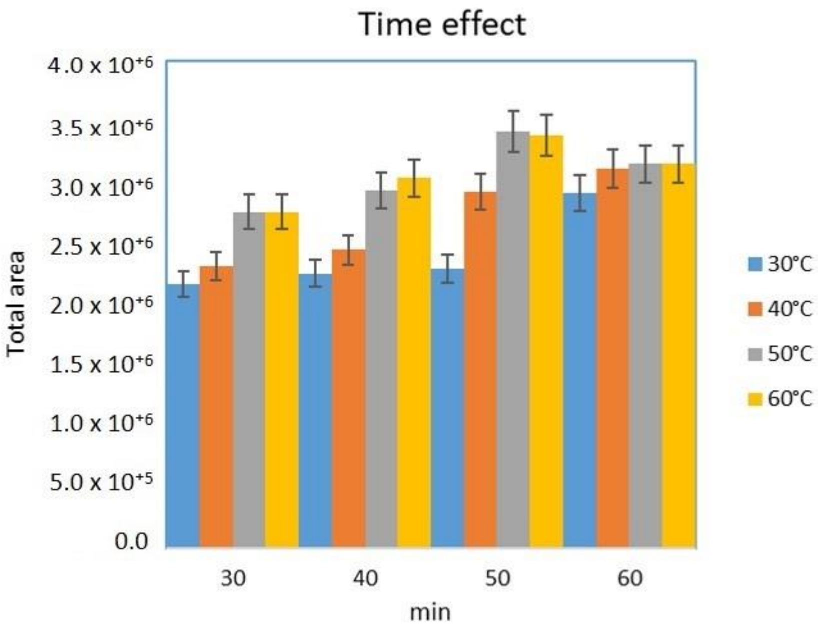

Figure S7. Influence of time on SPME method extraction optimization.

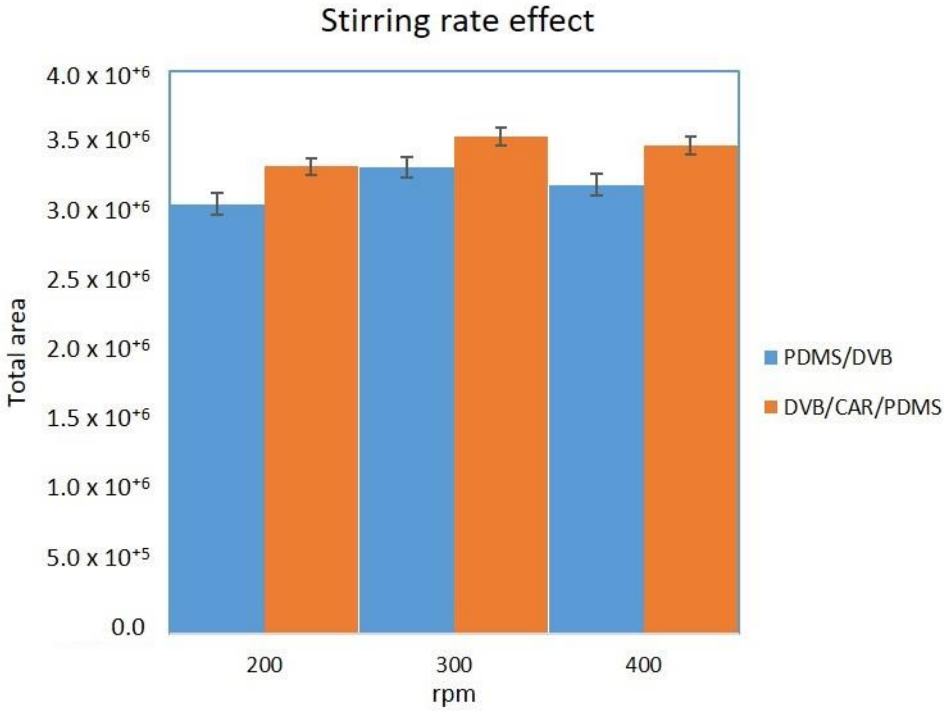

Figure S8. Influence of stirring rate on SPME method extraction optimization.

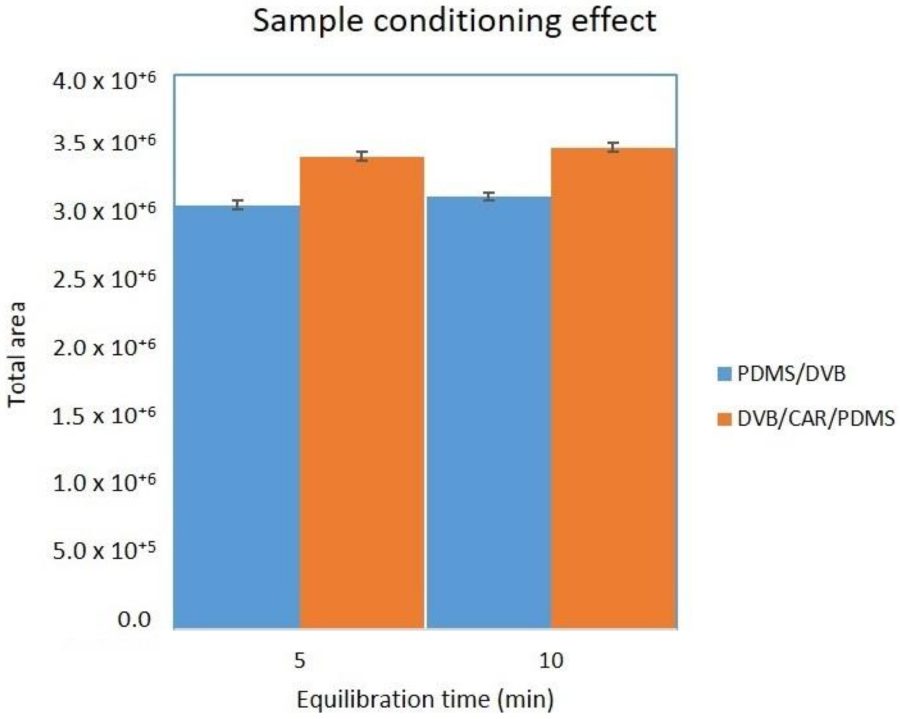

Figure S9. Influence of sample time conditioning on SPME method extraction optimization.

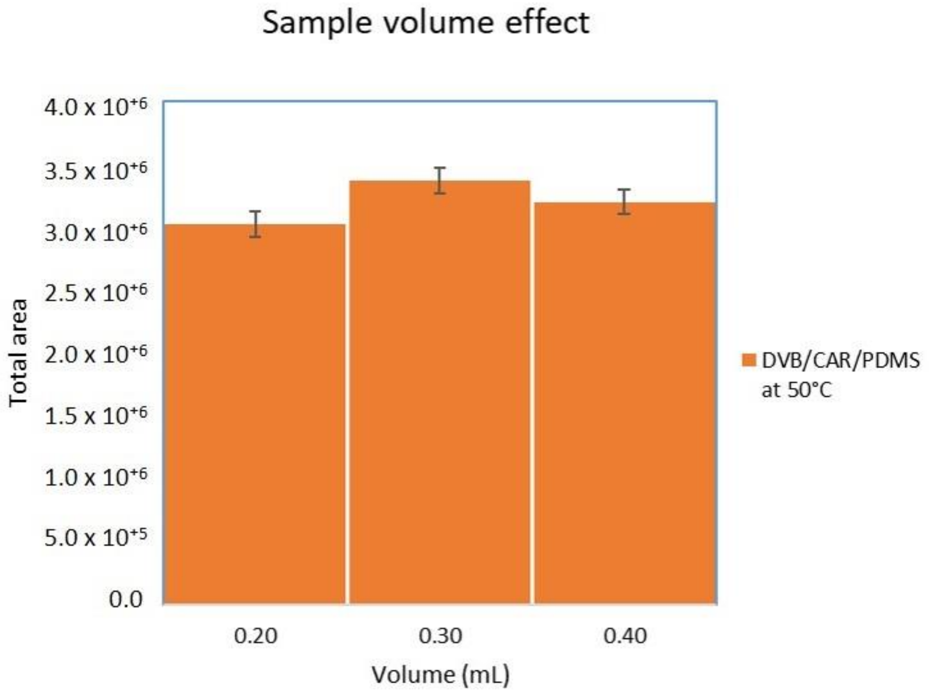

Figure S10. Influence of sample volume on SPME method extraction optimization.

Chili pepper sensorial analysis	
Fresh Fruity and Floral Notes	Fruity
	Apricot
	Watermelon
	Melon
	Banana
	Cherry
	Yellow apple
	green apple
	Pear
	Kiwi
	Citrus fruits
	Grapefruit
	Tropical fruit
	Pineapple
	Mango
	Litchi
	Floral
	Geranium
	Marigold
	Pink
	Iris - Violet
Fresh Vegetable Notes	Grass cut
	Galbanum
	Boxwood
	Artichoke
	Kale
	Thistle
	Cucumber
	Rue
	Tomato leaf
	Ripe pepper
	Green pepper
	Irritating
	Mustard, pungent
	Horseradish (Cren)
	Ginger
	Rapanelli
Dry Vegetable Notes	Paprika
	Dried tomato
	Hay, dry grass
	Black tea
	Walnut husk
	Dry leaves
	Tobacco
Other notes	Brine
	Smoked
	Fat green, oily
	Spicy
	Cinnamon
	Coriander
	Bitter almond
	Vanilla
	Chocolate
	Gaultheria
	Aromatic wood
	Caramel
	Fruit in syrup
	Concentrated tomato
	Fermented
	Dairy, cream, milk
	Blue cheese

Figure S11. List of descriptors used in the sensory analysis of chili peppers
